# Cancer pain assessment and management: does an institutional approach individualise and reduce cost of care?

**DOI:** 10.1136/spcare-2022-003547

**Published:** 2023-05-26

**Authors:** Katharina Diernberger, Eleanor Clausen, Gordon Murray, Bee Wee, Stein Kaasa, Peter Hall, Marie Fallon

**Affiliations:** 1 University of Edinburgh Western General Hospital, Edinburgh, UK; 2 The International Spine Centre, Adelaide, South Australia, Australia; 3 Public Health Sciences, University of Edinburgh, Edinburgh, UK; 4 University of Oxford, Oxford, UK; 5 University of Oslo Faculty of Medicine, Oslo, Norway; 6 Department of Palliative Medicine, Western General Hospital, University of Edinburgh, Edinburgh, UK

**Keywords:** Cancer, Pain, Clinical assessment, Service evaluation, Supportive care

## Abstract

**Objectives:**

To understand individual prescribing and associated costs in patients managed with the Edinburgh Pain Assessment and management Tool (EPAT).

**Methods:**

The EPAT study was a two-arm parallel group cluster randomised (1:1) trial, including 19 UK cancer centres. Study outcome assessments, including pain levels, analgesia and non-pharmacological and anaesthetic interventions, collected at baseline, 3–5 days and, if applicable, 7–10 days after admission. Costs calculated for inpatient length of stay (LoS), medications and complex pain interventions. Analysis accounted for the clustered nature of the trial design. In this post-hoc analysis, healthcare utilisation and costs are presented descriptively.

**Participants:**

10 centres randomised to EPAT (487 patients) and 9 (449 patients) to usual care (UC).

**Main outcome measures:**

Pharmacological and non-pharmacological management, complex pain interventions, length of hospital stay and costs related to these outcomes.

**Results:**

The mean per patient hospital cost was £3866 with EPAT and £4194 with UC, reflecting a mean LoS of 2.9 days and 3.1 days, respectively. Costs were lower for non-opioids, Non-steroidal anti-inflammatories (NSAIDs) and opioids but slightly higher for adjuvants with EPAT than with UC. The mean per-patient opioid costs were £17.90 (EPAT) and £25.80 (UC). Mean per patient costs of all medication were £36 (EPAT) and £40 (UC).

Complex pain intervention costs were £117 with EPAT per patient and £90 with UC. Overall mean cost per patient was £4018.3 (95% CI 3698.9 to 4337.8) with EPAT and £4323.8 (95% CI 4060.0 to 4587.7) with UC.

**Conclusions:**

EPAT facilitated personalised medicine and may result in less opioids, more specific treatments, improved pain outcomes and cost savings.

**What was already known?**

- Edinburgh Pain Assessment and management Tool (EPAT) resulted in improved pain relief with no increase in opioid related side effects.

**What are the new findings?**

- EPAT results in prescription of less opioids but more adjuvant analgesics and non-pharmacological approaches. Reduces costs overall.

**What is their significance?**

- Clinical
  Consistent simple language and linked algorithms improves pain management, individualises treatment and is cost efficient.
- Research
  Implementation science methodology is an important next step.

## Introduction

Pain is a common symptom among patients with cancer; moderate to severe pain is estimated to affect 38% of patients across all disease stages and 52% of patients with advanced, metastatic or terminal disease.^1^ The suboptimal management of cancer related pain and subsequent impact on quality of life, functioning, out of hours medical contact and tolerability of cancer treatment is well recognised.^2 3^ Simple analgesic strategies in patients with metastatic disease have been shown to lead to good pain control in 85% of patients.^4^ However, in the complex environment of modern cancer care, a lack of attention to basic pain assessment and management is common.^5^

We hypothesised that we could improve cancer pain outcomes in cancer centre inpatients by introducing, at institutional level, a simple systematic approach to pain assessment and management using the Edinburgh Pain Assessment and management Tool (EPAT). This new approach is based on strategies to deal with the common clinical shortcomings in cancer care. These include: (a) unstructured assessment, (b) use of treatment guidelines that lack explicit algorithms and do not address clinicians’ concerns about prescribing opioids and (c) lack of systematic monitoring of outcomes including adverse effects.^6 7^ EPAT facilitates improved assessment with an initial focus on the patient’s report of their worst pain, pharmacological management simplified by algorithms, reminder of non-pharmacological approaches and regular reassessment of pain and of drug side effects.

In a study of the effectiveness of EPAT, previously published in the *Journal of Clinical Oncology*,^8^ we compared the effect of EPAT (a policy of adding a brief clinician-delivered bedside pain assessment and management tool to usual care (UC)), with that of UC alone, on pain and prescribing outcomes. EPAT achieved better outcomes in both pain and in prescribing practice. In addition, while there was no increase in either opioid prescribing nor in opioid related side effects as a result of EPAT, there was an improvement in how opioids were prescribed.

However, we still need to know (a) whether EPAT leads to the use of more complex interventions and (b) whether it leads to a shift from cheap opioid analgesics to more expensive adjuvant analgesics. While this was not a defined outcome of the EPAT study, data were collected that allows post-hoc analysis. The aim of this analysis, therefore, was to examine the components of pain management in each patient in the EPAT study and the costs incurred by these components.

## Methods

We used data from the EPAT study, a two-arm parallel group cluster randomised (1:1) trial, in which we recruited consecutive patients on admission with a worst pain score in the last 24 hours of at least 4 out of 10, and observed pain outcomes with UC in 19 UK cancer centres. Randomisation of centres took into account size of centre and location. After the UC phase, we then randomised centres to either implement EPAT or to continue UC. The primary outcome was change in the percentage of study participants in each centre with a clinically significant (≥2 point) improvement in ‘worst pain’ (Brief Pain Inventory Short Form, BPI-SF) from admission to 3–5 days after admission. Secondary outcomes included quality of analgesic prescribing and opioid-related side effects. Data on additional management of pain and hospital stays were captured prospectively in the study but was not predefined as an outcome measure.

Ten centres were randomised to EPAT and nine to UC. We enrolled 1921 patients overall and obtained outcome data from 93% (1795); however, we include here just the patient data obtained after the centres were randomised into the intervention phase. Participants (mean age: 60 years, 49% female) had a variety of cancer types.

### Study assessments

All study outcome assessments, including, BPI, analgesia prescribed, non-pharmacological and anaesthetic interventions, were completed by an independent research nurse at baseline (within 24 hours of admission), 3–5 days after admission and for those who were still an inpatient, at day 7 and day 10 after admission. Date of discharge was collected for all patients. Full details of the study can be found in the published paper and in the protocol.^8 9^

### Delivery of pain management in the study

The clinicians who delivered pain assessment and management (EPAT or UC) were oncology nurses, general nurses, junior doctors, oncology trainee doctors and oncologists. In UC centres, the clinical team managed patients according to their existing knowledge, attitudes and local pain guidelines. In EPAT centres, the clinical team was provided with the pain assessment and management tool (online supplemental appendix 1) and given brief (30–60 min) group training in its use. This training was integrated with the existing educational and update forum structure in each centre.

10.1136/spcare-2022-003547.supp1 Supplementary data

The training could be very short, as the tool contains no new knowledge, rather it provides a structure and system for the existing general knowledge among professionals working with cancer patients.

### Data on costs

Costs were calculated for:

- The length of stay (LoS) within the hospital.
- The medication costs according to the prespecified frequencies and mode of administration.
- The intervention costs were calculated for complex pain interventions, such as spinal analgesia, non-pharmacological interventions such as transcutaneous electrical nerve stimulation (TENS), and palliative radiotherapy for pain.

All costing data, as well as data used for the descriptive statistics, were truncated at day 7 in order to stay in line with the original study design. For those patients where the entry date was equal to the discharge date, the day count was set to 0 which meant that their LoS was 0 days, nevertheless all other costs (medication and intervention) were included in the analysis.

Costs were calculated by the assignment of unit costs to observed resource use and activity. Unit costs were obtained from standard UK NHS reference sources, including NHS reference costs as well as Personal Social Services Research Unit (PSSRU) cost. Costs were assigned to LoS using a per-diem cost of £745 per day (PSSRU 2018). Units of resource and unit costs are presented in online supplemental appendix 2.

10.1136/spcare-2022-003547.supp2 Supplementary data

### Statistical analysis

Descriptive statistics split by randomised group (EPAT vs UC) were produced for all complex pain interventions as well as non-pharmacological interventions. Further descriptive statistics show the medication costs categorised in the same way as in the original trial design, namely, non-opioids, weak opioids, strong opioids, adjuvant analgesics, non-steroidal anti-inflammatories and others. Ninety-five per cent CIs around mean costs were calculated by bootstrapping.

All analysis took into account the clustered nature of the trial design. Mean values were first summarised within centres and then the within centre averages were averaged over centres.

The preparation of the costs was done in Excel. The main analysis as well as data cleaning were performed using the free and open-source programming language R (R Studio V.3.6.1).

## Results

Baseline characteristics are similar for the EPAT and UC group for pain (worst pain and BPI-SF) and distress scores, with a fairly even split between gender in both groups and a mean age of 59.4 years and 59.7 years, respectively (table 1).

**Table 1:** Participant baseline characteristics

Characteristic	EPAT (487)		UC (449)	
	Mean	%	Mean	%
Sex				
Female	240	49.3%	218	48.6%
Male	247	50.7%	231	51.4%
Age				
Years	59.4	13.1(SD)	59.7	13.2 (SD)
Primary cancer				
Breast	64	13.1%	42	9.4%
Genitourinary	69	14.2%	73	16.3%
Gynaecological	35	7.2%	37	8.2%
GI	69	14.2%	56	12.5%
Lung	49	10.1%	48	10.7%
Head and neck	38	7.8%	45	10.0%
Haematological	41	8.4%	21	4.7%
Other	115	23.6%	113	25.2%
Unknown	5	1.0%	14	3.1%
Baseline scores				
Worst pain	7.8	1.8 (SD)	7.8	1.9 (SD)
Global distress	6.4	3.0 (SD)	6	3.2 (SD)
Total BPI-SF	5.7	1.8 (SD)	5.6	1.9 (SD)

BPI-SF, Brief Pain Inventory Short Form; EPAT, Edinburgh Pain Assessment and management Tool; GI, Gastro-instestinal; UC, usual care.

### LoS split by randomisation group

**Figure 1:**
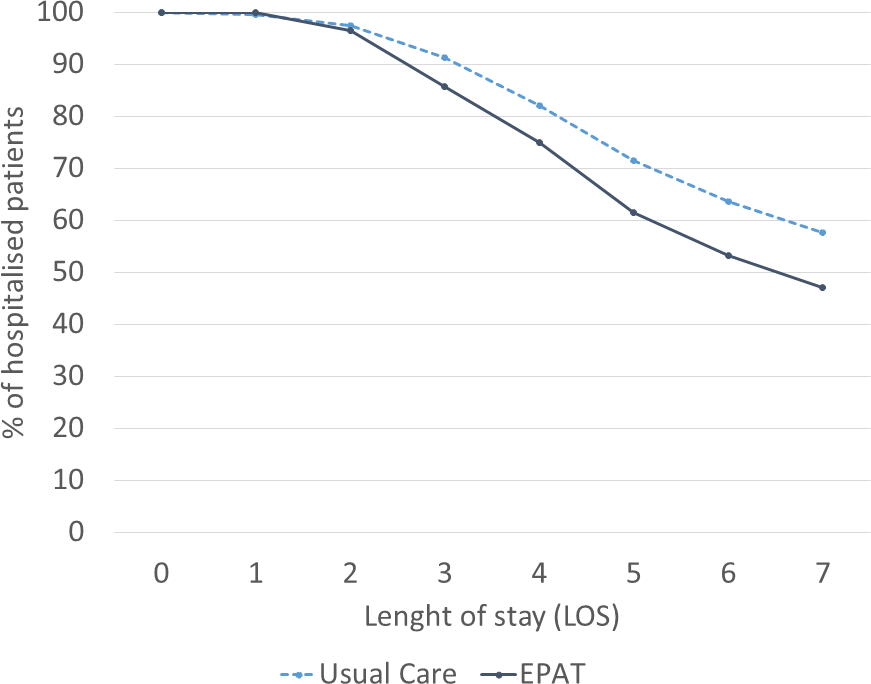
LoS split by randomisation group. EPAT, Edinburgh Pain Assessment and management Tool; LoS, length of stay.

At the start of the intervention phase of the study, 487 patients were recruited and randomised into the EPAT group and 449 patients into the UC group at day 0. From day 2 onwards, the percentage of patients who were still hospitalised was lower in the EPAT than the UC group (figure 1). On the last day included in the analysis 47% in the EPAT group were still in hospital compared with 57.6% of the patients in the UC group. The mean LoS was 2.9 days in the EPAT group compared with 3.1 days in the UC group.

### Costs for inpatient time (LoS), medication and pain interventions

The biggest driver of the costs in both arms was the LoS, with an average cost per patient of £3866 in the EPAT group and £4194 in the UC group (table 2). This reflects the results shown in figure 1 where it can be observed that earlier discharge is more frequent in the EPAT group.

**Table 2:** Per patient costs for the LoS, medication and interventions

Costs (GBP £)	UC (mean) (95% CI)	EPAT (mean) (95% CI)
LoS	4193.7 (3954.4 to 4433.0)	3865.7 (3554.9 to 4176.5)
Medication	40.3 (20.4 to 59.8)	35.8 (22.3 to 49.3)
Intervention	89.8 (52.2 to 127.4)	116.7 (54.4 to 179.0)
Total	4323.8 (4060.0 to 4587.7)	4018.3 (3698.9 to 4337.8)

EPAT, Edinburgh Pain Assessment and management Tool; LoS, length of stay; UC, usual care.

The medication costs were slightly lower in the EPAT group at £36 per patient compared with £40 per patient in the UC group. The pain specific intervention costs were £117 in the EPAT group and £90 in the UC group. These are ‘one off’ procedure costs. Patients in the EPAT group were more likely to receive highly specific, tailored analgesic procedures as seen in tables 3 and 4.

**Table 3:** Complex pain interventions by randomisation

Timepoint	LNB	CPB	EIT	NPI
Total UC	4 (0.88%)	2 (0.44%)	3 (0.66%)	0 (0.0%)
Total EPAT	21 (4.41%)	3 (0.61%)	28 (5.75%)	2 (0.41%)

CPB, coeliac plexus block; EIT, external intrathecal; EPAT, Edinburgh Pain Assessment and management Tool; LNB, local nerve block; NPI, neurosurgical pain interventions; UC, usual care.

**Table 4:** Non-pharmacological interventions

Time point	TENS	Acupuncture	Heat	Cold	CAM
Total UC	23	20	149	51	103
Total EPAT	68	33	180	67	79

CAM, complementary and alternative medicine; EPAT, Edinburgh Pain Assessment and management Tool; TENS, transcutaneous electrical nerve stimulation; UC, usual care.

### Individualised complex pain interventions and non-pharmacological interventions

Patients assessed by the EPAT tool were more likely to receive complex pain interventions than patients in the UC group (table 3). Of note, an external intrathecal line was inserted in 18 patients in the EPAT group, and in only 2 in the UC group.

The results from the non-pharmacological interventions follow the same trend, showing that all of the interventions were more commonly used in the EPAT group than the UC group. An example of this is the cheap and simple transcutaneous electrical nerve stimulation (TENS) treatment; this was received by 68 patients in the EPAT group compared with only 23 patients in the UC group.

### Radiotherapy for uncontrolled pain

Radiotherapy is used as standard management of some types of cancer pain, especially cancer induced bone pain. Table 4 shows that only 30% of patients in the EPAT group required referral for radiotherapy, while 40% of patients in the UC group required referral for radiotherapy.

### Medication costs split up into different drug groups

The mainstay of pharmacological management of cancer pain is based on the following drug groups: non-opioids, weak opioids, strong opioids, non-steroidal anti-inflammatories (NSAIDs) and adjuvant analgesics. Table 5 shows the costs for these key drug groups of interest in cancer pain split by the EPAT and UC groups. Results are presented as overall costs and broken down into costs per patient. The first three lines show the costs for non-opioids, adjuvants and NSAIDs. Looking at the costs broken down on a patient level for EPAT and UC groups, 487 and 453, respectively, the costs for these non-opioid drugs are small compared with the opioid costs. In the EPAT group, there are lower costs for non-opioids and NSAIDs and slightly higher costs for adjuvants compared with the UC group. Looking at the opioid costs, it can be seen that the costs for both weak and strong opioids are lower in the EPAT group compared with the UC group, with an overall per patient cost of £17.90 in the EPAT and £25.80 in the UC groups.

**Table 5:** Medication costs (in £ Sterling) for specific drug groups for control and EPAT group

Drug group	UC (total)	UC (mean)	EPAT (total)	EPAT (mean)
Non-opioids	195.1	0.4	167.8	0.3
Adjuvants	118.9	0.2	149.6	0.3
Non-steroidal anti-inflammatories	143.8	0.3	122.6	0.2
Weak opioids	265.7	0.5	148.9	0.3
Strong opioids	11 456.4	25.2	8581.4	17.6
Total opioids	11 722.2	25.8	8730.4	17.9

EPAT, Edinburgh Pain Assessment and management Tool; UC, usual care.

Table 5 shows that the EPAT group are prescribed more adjuvant analgesics but do not have a higher use of any other medication, especially of weak or strong opioids.

There were no regional differences for use of non-pharmacological interventions.

## Discussion

In the main EPAT study, already published, we demonstrated that improved pain relief was not associated with any excess of opioid related side effects.^8^ The aim of this analysis was to examine the components of pain management in each arm of the study and the costs incurred by these components.

This analysis shows that EPAT managed patients received less opioids, but more adjuvant analgesics, compared with UC. In addition, more non-pharmacological treatments, such as TENS machines, were used in the EPAT groups than UC. Similarly, the use of anaesthetic interventional techniques, although small numbers, was more common in the EPAT than the UC groups.

Of interest, fewer patients in the EPAT group required referral for palliative radiotherapy for pain control compared with the UC group.

These components of pain management point to improved pain relief with EPAT as a result of more appropriate individualised management. Adjuvant analgesic prescribing is a very good example of individualised care and implies that the nature of the cancer pain was assessed sufficiently to allow choice of this drug group. In spite of more individualised management, the overall analgesic costs were lower in the EPAT group, driven by less opioid use.

As is often seen in cost analyses, the major driver of costs is length of hospital stay. The EPAT group had a shorter hospital stay than the UC group and this was the major component of overall lower costs in the EPAT group.

In summary, EPAT led to improved pain relief, with less opioid; the opioid reduction was facilitated by more individualised pain management with resultant use of more targeted adjuvant analgesics and non-pharmacological pain interventions. The resultant shortened length of hospital stay drove an overall lower cost with EPAT than with UC.

### Controversies about opioid use

It is important to discuss EPAT within the context of the significant controversies which have evolved around pain assessment and management, particularly in the USA.

Many researchers have not been able to demonstrate improved pain treatment or better pain outcome by measuring pain as the fifth vital sign using numerical pain scores.^10–12^ As a result, there is a movement within the USA to abolish pain scores as a surrogate outcome measure of good care, and to stop the exclusive use of unidimensional pain assessment tools, as well as ending the direct relationship between provider reimbursement and patient self-reports of pain control.^13–19^ The Joint Commission, which acts as the regulatory body for many US healthcare institutions, now recognises that there is a direct link between healthcare policies, the numerical pain scale, pain expectations and opioid addiction.^8^ In an effort to mitigate against the harm from prescribed opioid addiction, the Joint Commission has developed 19 different ‘elements of performance’ (Eps) that accredited hospitals needed to comply with by January 2018.^19^ Eps 7 states that ‘using numerical pain scales (NPS) alone to monitor patients’ pain is inadequate’ and ‘stresses the importance of assessing how pain affects function and the ability to make progress towards treatment goals’.

The American Medical Association, the American College of Surgeons, the Joint Commission, The American Academy of Family Physicians and the Centres for Medicare and Medicaid services have all withdrawn their advocacy of the ‘pain as the 5th vital sign’ campaign. Unidimensional self-reported pain scores have been implicated in contributing to the prescribed opioid epidemic and also associated with over-sedation in the acute pain setting.^20^

### Personalised pain control

The further analyses presented in this paper provide insights into how a brief analytical assessment and linked management tool developed for use in the cancer inpatient setting is superior to a unidimensional pain rating scale and guidelines. EPAT establishes not just severity of pain, but the characteristics of pain which are then linked with management algorithms. The finding that more individualised and specific management strategies were used might help us to understand how EPAT can improve pain relief without any increase in opioid prescribing or increase in opioid-related side effects.

### Practical implications

It can be argued that the non-specialist clinician is empowered to prescribe more appropriately by directive assessment and linked algorithms, rather than a simple unidimensional pain scale and broad guidelines which simply suggest drug groups.

Cancer pain can be a complex construct and assessment of its many domains should be conducted using multidimensional tools. Many pain and symptom assessment tools exist for use in the cancer patient, including the BPI, the McGill Pain Questionnaire, the MD Anderson Symptom Inventory and the Edmonton Symptom Assessment System, among others. Our challenge is to have an appropriate bedside tool linked to appropriate management for consistent use by non-specialists.^21 22^

### Limitation

It is important to note that the cost analysis described in this paper was post hoc, meaning that it was not preplanned in the original EPAT trial protocol. Collection of healthcare utilisation was of sufficient depth and quality to make subsequent analysis of high value for decision-makers and policy. Comparative cost analysis is typically characterised by very high variance and skewed distributions, making sample size requirements for statistical comparisons very high and often not feasible in the context of a randomised controlled trial. For this reason, our analysis was entirely descriptive and we specifically avoided drawing conclusions of causal inference. Despite this, we believe that the conclusions of the study on the varying distribution of healthcare utilisation and costs between EPAT and UC as valid and likely represent the best achievable level of evidence in this challenging research context.

There are several additional specific limitations of our study was the focus only on inpatient management and costs. It is possible that earlier discharge may place a greater resource burden on community services. Further research is needed to understand the longer term post-discharge implications of EPAT and to assess the impact of an appropriately adapted community version on pain assessment and management, along with service use. Pain should have an appropriate and accessible common language for professionals, patients and carers and it needs to be consistent across all cancer care settings. The common language of, ‘pain as the 5th vital sign’, was too simple. We have demonstrated that institutionalisation of assessments appropriate to the clinical setting, linked with appropriate algorithms and reassessment, can both improve pain management with fewer opioids and save money. It is crucial for good patient care and a humane approach to pain, that we do not develop a fear around pain assessment, but rather develop more appropriate ways of assessing and managing pain.

## Conclusion

An institutional approach to standardise cancer pain assessment and management using EPAT led to less opioid prescribing and improved pain relief without side effects, via more individualised care with specific pharmacological and non-pharmacological management choices. In addition, EPAT also resulted in a cost saving.

These findings support the move away from pain assessment with a unidimensional tool and use of general guidelines and emphasise the importance of an assessment appropriate to the patient group which evaluates aetiology of pain, followed by individualised linked management and reassessment.

## Data Availability

Data are available upon reasonable request.
