# Supplementary material for: Cancer pain assessment and management: does an institutional approach individualise and reduce cost of care?
Source: BMJ Support Palliat Care. 2023 May 26;13(e3):e1258–64. doi: 10.1136/spcare-2022-003547 (PMC10850828; doi:10.1136/spcare-2022-003547)
Supplement: Supplementary data [file spcare-2022-003547supp001.pdf]

Edinburgh Pain Assessment Tool (EPAT©) VITAL SIGNS AND PAIN ASSESSMENT CHART – STEP 1

Ward Consultant

Surname First Names Age Unit No:

Date

Time

40⁰

39⁰ 230

220

38⁰ 210

200

37⁰ 190

180

36⁰ 170

160

35⁰ 150

140

34⁰ 130

120

BP

110

100

90

80

70

60

Pulse

50

40

30

20

10

Resps

Sp02

Score worst pain since last assessment 0 = No Pain 10 = Worst pain imaginable

PAIN 9-10

7-8

5-6

3-4

1-2

0

5-10 = Severe pain (act)
Give analgesia. Regular review until score is less than 3
Use EPAT© Step 2

3 – 4 = Moderate pain (act)
Give analgesia
Use EPAT© Step 2

0 = No pain
1-2 = Mild pain

NAUSEA SCORE

Nausea Score: 0 = No nausea 2 = Nausea (act) 3 = Vomiting (act) 4 = Nausea & vomiting (act)

Weight

Bowels

Other ………….

Edinburgh Pain Assessment Tool (EPAT ©) –Step 2

LOCATION

Name: Ward: Date/Time:

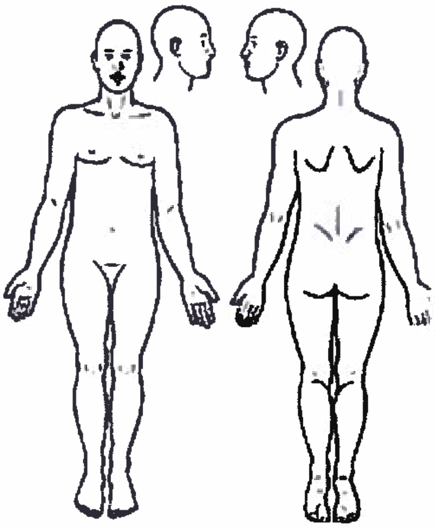

Severity Score	A Most Severe	B	C
Worst Pain in Last 24 hrs (0-10)			
Least Pain in Last 24 hrs (0-10)			

0 = No Pain 10 = Worst Pain Imaginable

On the diagram, mark the sites where you have felt worst pain (ABC)

Does your pain disturb your sleep?
Yes ☐ No☐

NERVE PAIN?

Is your pain...?

Shooting or Stabbing ☐ Tingling or Pricking ☐ Pins & Needles☐ Hot or burning ☐

➢

Do any areas of your skin feel numb or strange or unpleasant to touch?
Yes ☐ No ☐ Detail: ____________

Does moving or any other activity make your pain worse? Yes ☐ No☐

Does your pain come on suddenly at rest? Yes ☐ No☐

Consider neuropathic pain. **Treat** with adjuvants
Use 'starting gabapentin/starting amitriptyline' EPAT algorithms

OVEMENT PAIN?

Does moving or any other activity make your pain worse? Yes ☐ No☐

Does your pain come on suddenly at rest? Yes ☐ No☐

Is the patient experiencing movement-related or spontaneous pain? Consider bone pain
Use WHO analgesic ladder – See EPAT algorithm. **Give** PRN analgesia before movement
Consider NSAID's / Palliative Radiotherapy / Bisphosphonates

WIDER ISSUES

What makes your pain better? ____________

Remember: non-pharmacological interventions!
Consider: Position change / Relaxation / Physiotherapy/ Rubbing / TENS / acupuncture

☹ **Is there anything worrying or concerning you about your pain?** Discuss with patient.
Remember: ☹ anxiety/depression may co-exist with severe pain.

REFERRAL

Consider referral to your Specialist Team for patients who have persistent pain:

- ➢Patients with severe pain
- ➢Patients requiring a rapidly increasing opioid dose
- ➢Patients with opioid-induced drowsiness
- ➢Patients with movement-related pain
- ➢Pain unrelieved by initial management

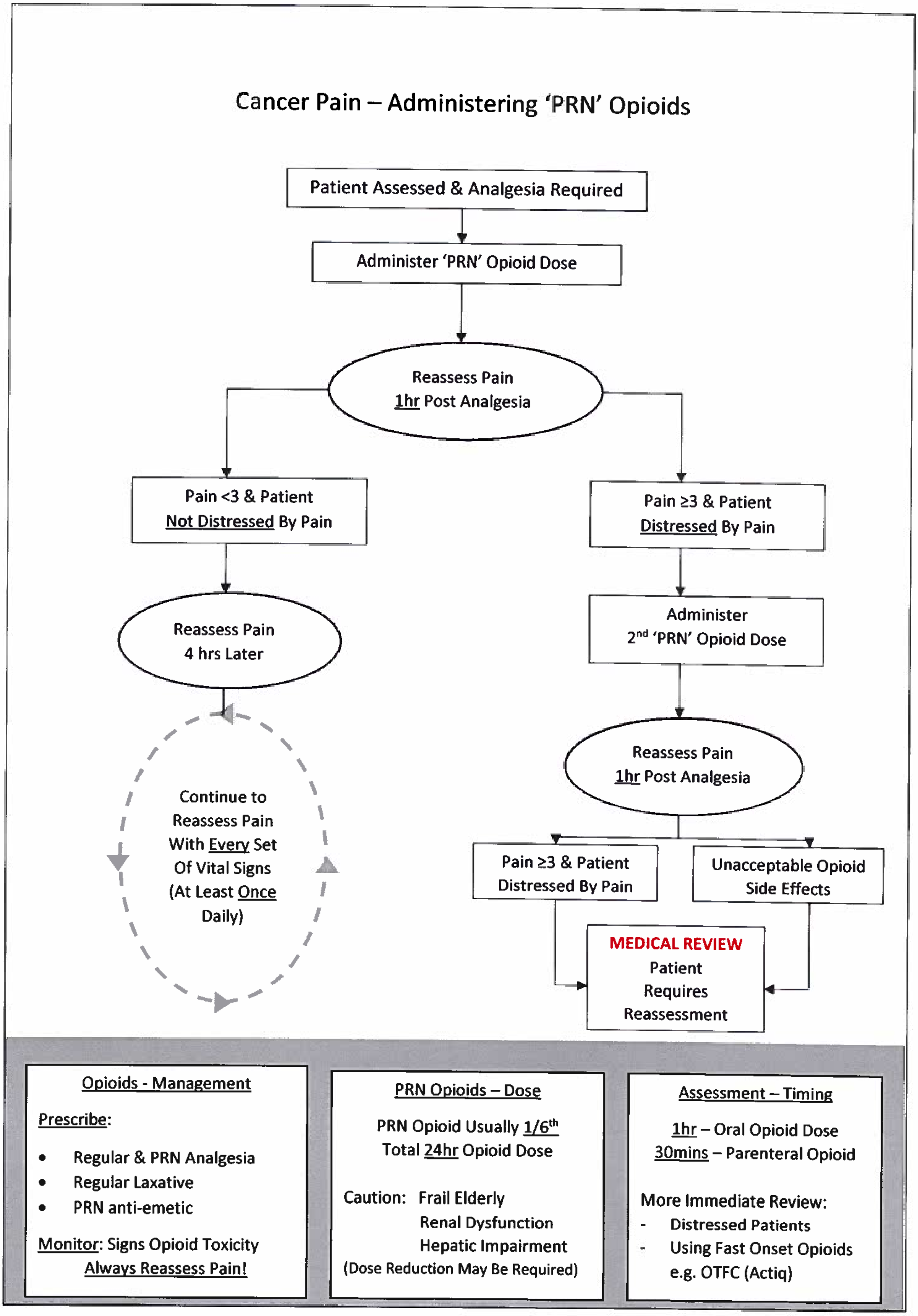
Cancer Pain – Administering 'PRN' Opioids
Patient Assessed & Analgesia Required
Administer 'PRN' Opioid Dose
Reassess Pain 1hr Post Analgesia
Pain <3 & Patient Not Distressed By Pain
Reassess Pain 4 hrs Later
Continue to Reassess Pain With Every Set Of Vital Signs (At Least Once Daily)
Pain ≥3 & Patient Distressed By Pain
Administer 2nd 'PRN' Opioid Dose
Reassess Pain 1hr Post Analgesia
Pain ≥3 & Patient Distressed By Pain
Unacceptable Opioid Side Effects
MEDICAL REVIEW
Patient Requires Reassessment
Opioids - Management
Prescribe:
• Regular & PRN Analgesia
• Regular Laxative
• PRN anti-emetic
Monitor: Signs Opioid Toxicity
Always Reassess Pain!
PRN Opioids – Dose
PRN Opioid Usually 1/6th Total 24hr Opioid Dose
Caution: Frail Elderly
Renal Dysfunction
Hepatic Impairment
(Dose Reduction May Be Required)
Assessment – Timing
1hr – Oral Opioid Dose
30mins – Parenteral Opioid
More Immediate Review:
- Distressed Patients
- Using Fast Onset Opioids e.g. OTFC (Actiq)

Edinburgh Pain Assessment Tool (EPAT©) – Treatment Review Chart

Name: ______________ DOB: ☐☐☐☐☐☐ Unit/Hospital No: ______________ Ward: ______________

**Date & Time**						
**Worst Pain Now**	(0-10) ______	(0-10) ______	(0-10) ______	(0-10) ______	(0-10) ______	(0-10) ______
**Worst Pain Site** (specify)	______	______	______	______	______	______
**Pain Distress**	Is your pain distressing:<br>☐Yes ☐No<br>Comment:<br>______<br>______	Is your pain distressing:<br>☐Yes ☐No<br>Comment:<br>______<br>______	Is your pain distressing:<br>☐Yes ☐No<br>Comment:<br>______<br>______	Is your pain distressing:<br>☐Yes ☐No<br>Comment:<br>______<br>______	Is your pain distressing:<br>☐Yes ☐No<br>Comment:<br>______<br>______	Is your pain distressing:<br>☐Yes ☐No<br>Comment:<br>______<br>______
**Opioid toxicity**<br>**Stop & Check Daily!**	☐Shadows-corner of your eyes<br>☐ Drowsiness ☐Vivid dreams<br>☐Hallucinations ☐Confusion<br>☐Jerking/twitching<br>Check-opioid dose may not suit	☐Shadows-corner of your eyes<br>☐ Drowsiness ☐Vivid dreams<br>☐Hallucinations ☐Confusion<br>☐Jerking/twitching<br>Check-opioid dose may not suit	☐Shadows-corner of your eyes<br>☐ Drowsiness ☐Vivid dreams<br>☐Hallucinations ☐Confusion<br>☐Jerking/twitching<br>Check-opioid dose may not suit	☐Shadows-corner of your eyes<br>☐ Drowsiness ☐Vivid dreams<br>☐Hallucinations ☐Confusion<br>☐Jerking/twitching<br>Check-opioid dose may not suit	☐Shadows-corner of your eyes<br>☐ Drowsiness ☐Vivid dreams<br>☐Hallucinations ☐Confusion<br>☐Jerking/twitching<br>Check-opioid dose may not suit	☐Shadows-corner of your eyes<br>☐ Drowsiness ☐Vivid dreams<br>☐Hallucinations ☐Confusion<br>☐Jerking/twitching<br>Check-opioid dose may not suit
**Intervention** (specify)	☐Analgesia<br>☐ Non-Pharmacological<br>☐Other<br>______<br>______	☐Analgesia<br>☐ Non-Pharmacological<br>☐Other<br>______<br>______	☐Analgesia<br>☐ Non-Pharmacological<br>☐Other<br>______<br>______	☐Analgesia<br>☐ Non-Pharmacological<br>☐Other<br>______<br>______	☐Analgesia<br>☐ Non-Pharmacological<br>☐Other<br>______<br>______	☐Analgesia<br>☐ Non-Pharmacological<br>☐Other<br>______<br>______
**Outcome**<br>(Review 1 hr after analgesia)	**Worst pain (0-10)** ______<br>Is your pain distressing now:<br>☐Yes ☐No<br>______<br>______	**Worst pain (0-10)** ______<br>Is your pain distressing now:<br>☐Yes ☐No<br>______<br>______	**Worst pain (0-10)** ______<br>Is your pain distressing now:<br>☐Yes ☐No<br>______<br>______	**Worst pain (0-10)** ______<br>Is your pain distressing now:<br>☐Yes ☐No<br>______<br>______	**Worst pain (0-10)** ______<br>Is your pain distressing now:<br>☐Yes ☐No<br>______<br>______	**Worst pain (0-10)** ______<br>Is your pain distressing now:<br>☐Yes ☐No<br>______<br>______
**Next Review** (Date & Time)						

Analgesic Interventions
- Breakthrough Analgesia
- ↑Dose Regular Analgesia
- Regular Analgesia Changed
- Adjuvant Drug Prescribed
- ↑Adjuvant Dose

Non-Pharmacological Interventions
- Position change
- Physiotherapy
- Heat
- Cold
- TENS
- Massage
- Acupuncture
- Relaxation

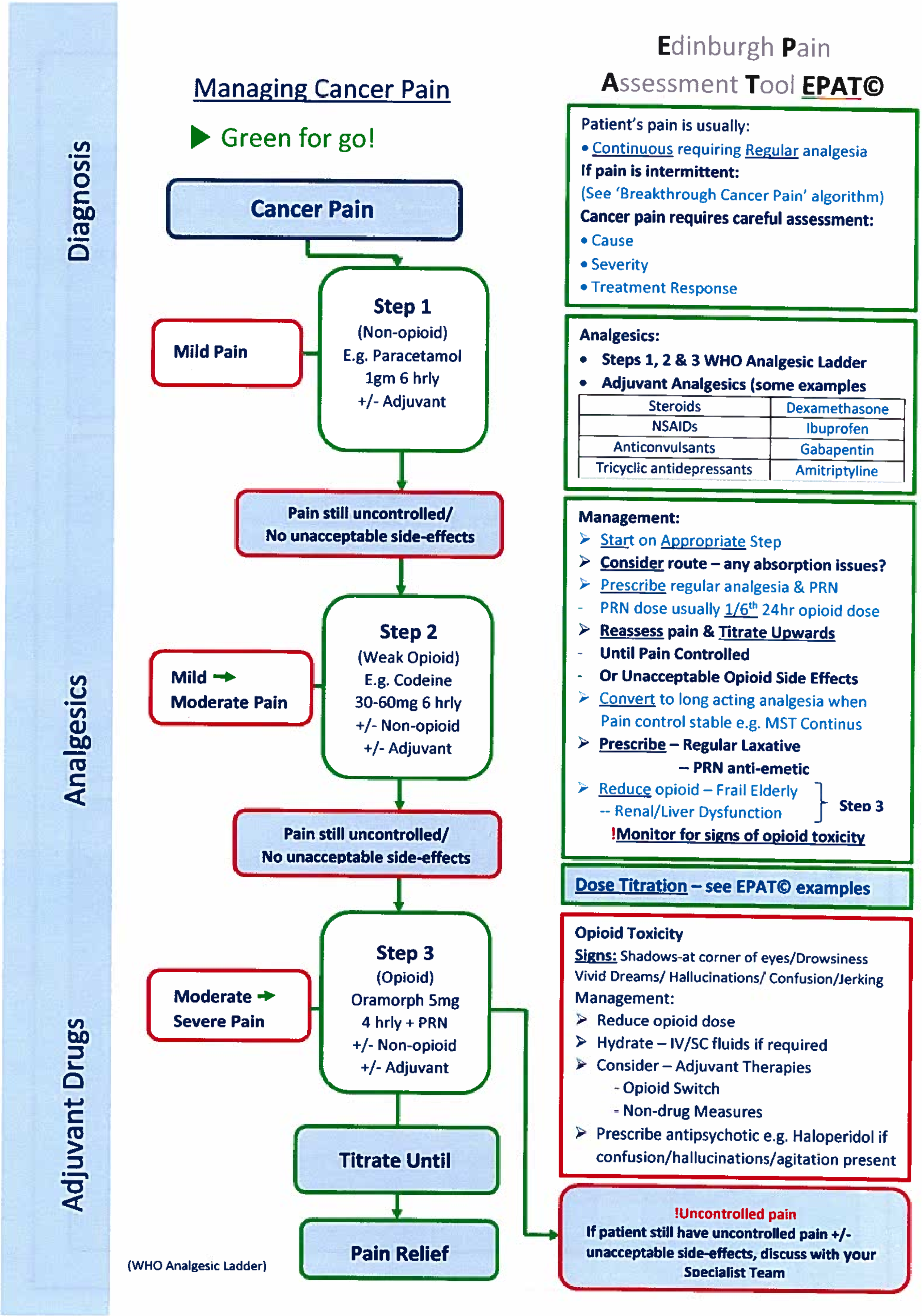
Diagnosis
Analgesics
Adjuvant Drugs
Managing Cancer Pain
Green for go!
Cancer Pain
Mild Pain
Step 1
(Non-opioid)
E.g. Paracetamol
1gm 6 hrly
+/- Adjuvant
Pain still uncontrolled/
No unacceptable side-effects
Mild → Moderate Pain
Step 2
(Weak Opioid)
E.g. Codeine
30-60mg 6 hrly
+/- Non-opioid
+/- Adjuvant
Pain still uncontrolled/
No unacceptable side-effects
Moderate → Severe Pain
Step 3
(Opioid)
Oramorph 5mg
4 hrly + PRN
+/- Non-opioid
+/- Adjuvant
Titrate Until
Pain Relief
(WHO Analgesic Ladder)
Edinburgh Pain Assessment Tool EPAT©
Patient's pain is usually:
• Continuous requiring Regular analgesia
If pain is intermittent:
(See 'Breakthrough Cancer Pain' algorithm)
Cancer pain requires careful assessment:
• Cause
• Severity
• Treatment Response
Analgesics:
• Steps 1, 2 & 3 WHO Analgesic Ladder
• Adjuvant Analgesics (some examples
Steroids | Dexamethasone
NSAIDs | Ibuprofen
Anticonvulsants | Gabapentin
Tricyclic antidepressants | Amitriptyline
Management:
➢ Start on Appropriate Step
➢ Consider route – any absorption issues?
➢ Prescribe regular analgesia & PRN
- PRN dose usually 1/6th 24hr opioid dose
➢ Reassess pain & Titrate Upwards
- Until Pain Controlled
- Or Unacceptable Opioid Side Effects
➢ Convert to long acting analgesia when Pain control stable e.g. MST Continus
➢ Prescribe – Regular Laxative
– PRN anti-emetic
➢ Reduce opioid – Frail Elderly
-- Renal/Liver Dysfunction
Step 3
!Monitor for signs of opioid toxicity
Dose Titration – see EPAT© examples
Opioid Toxicity
Signs: Shadows-at corner of eyes/Drowsiness
Vivid Dreams/ Hallucinations/ Confusion/Jerking
Management:
➢ Reduce opioid dose
➢ Hydrate – IV/SC fluids if required
➢ Consider – Adjuvant Therapies
- Opioid Switch
- Non-drug Measures
➢ Prescribe antipsychotic e.g. Haloperidol if confusion/hallucinations/agitation present
!Uncontrolled pain
If patient still have uncontrolled pain +/- unacceptable side-effects, discuss with your Specialist Team

EPAT© Dose Titration Examples

Example 1 – Continuous Pain

Mr A is prescribed Oramorph 5 mg 4 Hourly and Oramorph 5 mg PRN for breakthrough pain – he has required 3 extra doses of Oramorph over the last 24 hours.
He describes his cancer-related pain as 'almost constant' and states the Oramorph is providing only partial pain relief – he has no apparent side-effects on assessment.

Titration – Oramorph 5 mg 4 hourly – 5 mg x 6 = 30 mg + PRN doses 5 mg x 3 = 15 mg

Total 24 Hr Dose Requirement = Oramorph 45 mg

New Titration Dose: Oramorph 45 mg ÷ 6 = 7.5 mg 4 Hourly & PRN dose 7.5 mg

Example 2 – Breakthrough Cancer Pain (End-of-dose-failure)

Mrs B is receiving Oramorph 4 Hourly and Oramorph 30 mg PRN for breakthrough pain – she is finding her pain is generally well controlled for approximately 3 hours then it returns. She is using 2 PRN doses for breakthrough pain daily with reluctance, as she feels drowsy if a PRN dose is required closely (within an hour) of her regular 4 Hourly dose.

Titration – Oramorph 30 mg 4 hourly – 30 mg x 6 = 180 mg + PRN doses 30 mg x 2 = 60 mg

Total 24 Hr Dose Requirement = Oramorph 240 mg

New Titration Dose: Oramorph 240 mg ÷ 6 = 40 mg 4Hourly & PRN dose 40 mg

!Remember: If end-of-dose-failure is problematic and PRN doses are not being utilised – gently titrate Oramorph dose (25-30%) and consider switching to long acting preparations

Example 3 – Breakthrough Cancer Pain (Incident Pain)

Mr C is prescribed Oramorph 80 mg 4 Hourly and Oramorph 80 mg PRN for breakthrough pain – he related that when lying down his pain is more controlled and he requires 2 doses of PRN Oramorph over the day. When he attempts to mobilise however his pain becomes very severe and he requires a further 2 or 3 PRN doses daily prior to mobilising with only partial effect.

Titration – Oramorph 80 mg 4 hourly – 80 mg x 6 = 480 mg + PRN doses 80 mg x 2 = 160 mg*

Total 24Hr Dose Requirement = Oramorph 640 mg

New Titration Dose: Oramorph 640 mg ÷ 6 (rounded to) 100 mg 4 Hourly & PRN dose 100 mg

***If all PRN doses are included in titration where pain is mainly Incident, it is highly likely that patients will experience unacceptable opioid side-effects**

➔Initially aim for comfort at rest

➔Then titrate PRN dose & consider other interventions to optimise Breakthrough Pain control

Managing Breakthrough Cancer Pain

▶ Green for go!

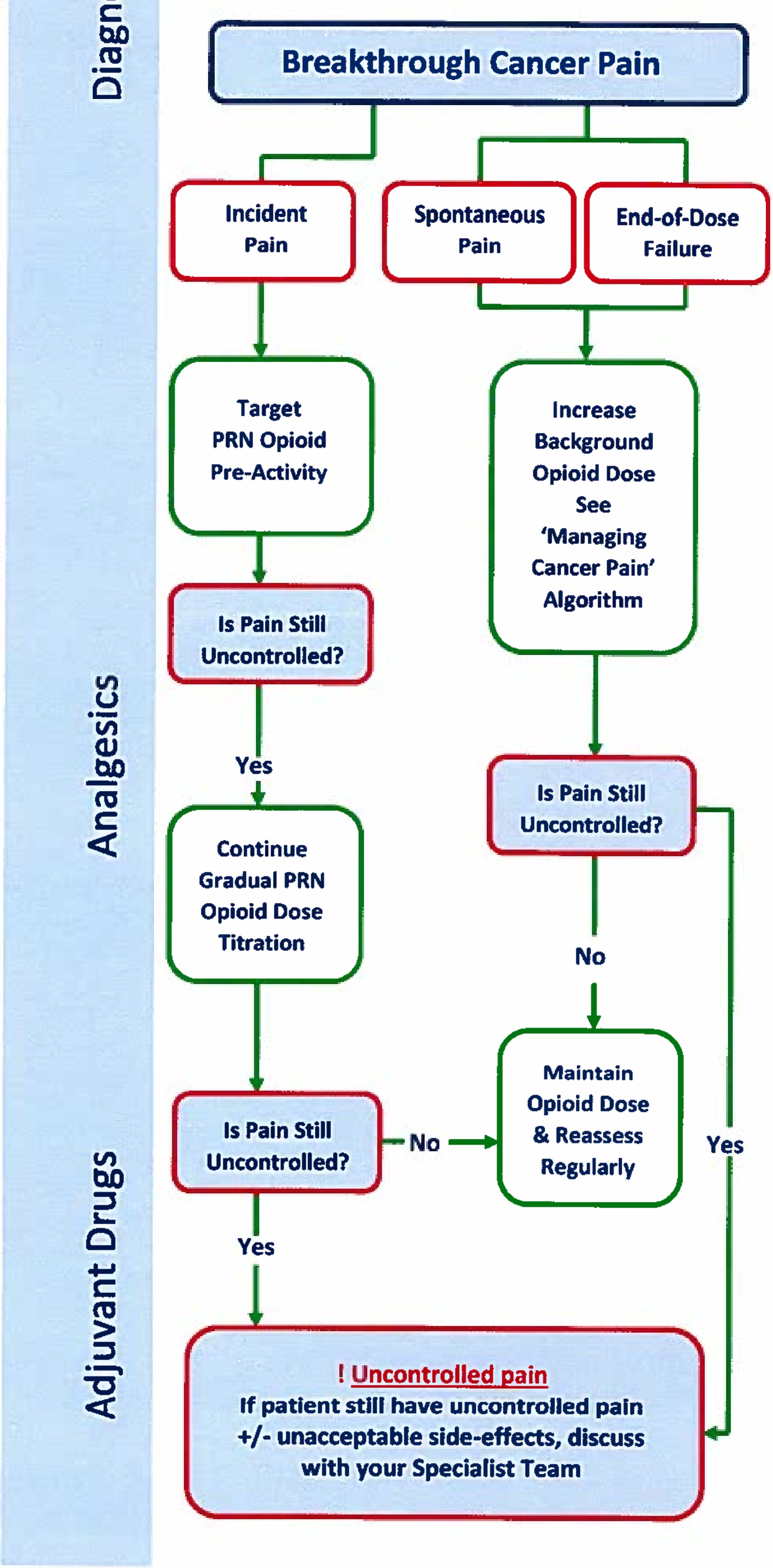

Edinburgh Pain Assessment Tool EPAT©

Breakthrough Cancer Pain (BTCP):
A transitory exacerbation of pain occurring on a background of adequately controlled baseline pain (Portenoy et al 2004).

Patient's pain is usually of:
- Sudden Onset
- Severe Intensity
- Short Duration (average 30 mins)

Breakthrough Pain Types:
- Incident: Related to activity e.g. Movement/ Micturition/ Coughing
- Spontaneous: Unexpected/At any time
- End-of-dose-failure: Insufficient Background Dose

Management:
- ➢ Incident Pain :
  Target PRN opioid dose Pre-Activity e.g. Movement-Related Pain
  - Oramorph 30 mins before mobilising
- ➢ Spontaneous Pain:
  Prescribe Immediate Release Opioid
  Increase background Opioid Dose
  Consider Adjuvant/(dose titration)
- ➢ End-of-dose-failure:
  Increase Background Opioid Dose

All Breakthrough Cancer Pain:
- ➢ Assess most appropriate Immediate Release Opioid (&Dose) for Patient
- ➢ Is Specialist Intervention Required?
- ➢ Consider Non-opioids & Adjuvants
- ➢ Consider Non-drug Measures
  - Heat/ TENS/ Activity Modification

! Stop titrating opioid dose if patient experiencing unacceptable side-effects

Analgesics:
- Steps 1, 2 & 3 WHO Analgesic Ladder & Adjuvant analgesics
- PRN Opioid Dose for BTCP – usually 1/6th 24hr total opioid dose
- Seek Specialist Advice
  - Pain is of very short duration
  - Side effects prevent ↑ PRN opioid

! Remember Breakthrough Cancer Pain is often associated with opioid toxicity

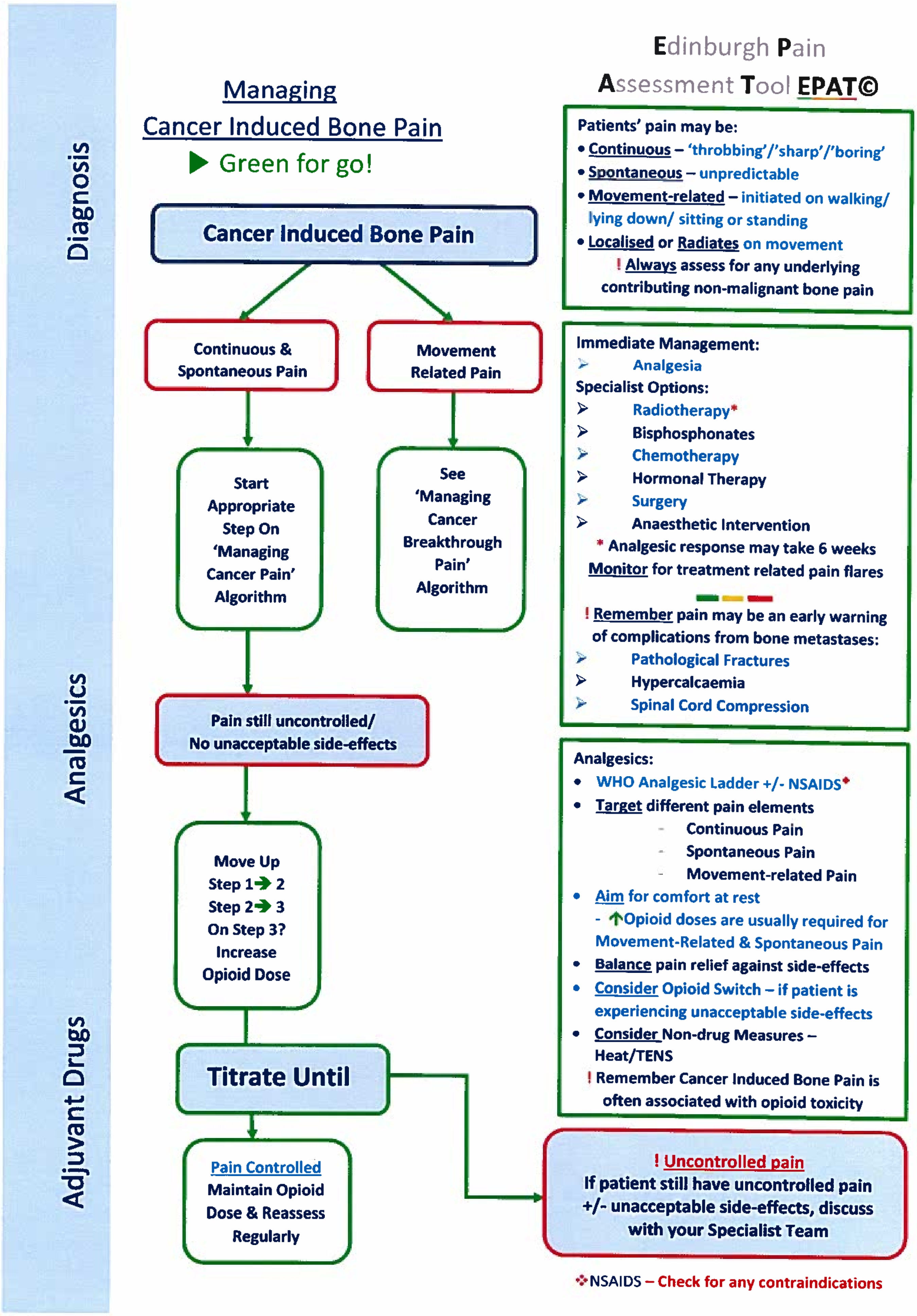
Diagnosis
Analgesics
Adjuvant Drugs
Managing
Cancer Induced Bone Pain
Green for go!
Cancer Induced Bone Pain
Continuous & Spontaneous Pain
Movement Related Pain
Start Appropriate Step On 'Managing Cancer Pain' Algorithm
See 'Managing Cancer Breakthrough Pain' Algorithm
Pain still uncontrolled/ No unacceptable side-effects
Move Up Step 1 → 2 Step 2 → 3 On Step 3? Increase Opioid Dose
Titrate Until
Pain Controlled Maintain Opioid Dose & Reassess Regularly
Edinburgh Pain Assessment Tool EPAT©
Patients' pain may be:
• Continuous – 'throbbing'/'sharp'/'boring'
• Spontaneous – unpredictable
• Movement-related – initiated on walking/ lying down/ sitting or standing
• Localised or Radiates on movement
! Always assess for any underlying contributing non-malignant bone pain
Immediate Management:
Analgesia
Specialist Options:
Radiotherapy*
Bisphosphonates
Chemotherapy
Hormonal Therapy
Surgery
Anaesthetic Intervention
* Analgesic response may take 6 weeks
Monitor for treatment related pain flares
! Remember pain may be an early warning of complications from bone metastases:
Pathological Fractures
Hypercalcaemia
Spinal Cord Compression
Analgesics:
• WHO Analgesic Ladder +/- NSAIDS*
• Target different pain elements
- Continuous Pain
- Spontaneous Pain
- Movement-related Pain
• Aim for comfort at rest
- ↑Opioid doses are usually required for Movement-Related & Spontaneous Pain
• Balance pain relief against side-effects
• Consider Opioid Switch – if patient is experiencing unacceptable side-effects
• Consider Non-drug Measures – Heat/TENS
! Remember Cancer Induced Bone Pain is often associated with opioid toxicity
! Uncontrolled pain
If patient still have uncontrolled pain +/- unacceptable side-effects, discuss with your Specialist Team
NSAIDS – Check for any contraindications

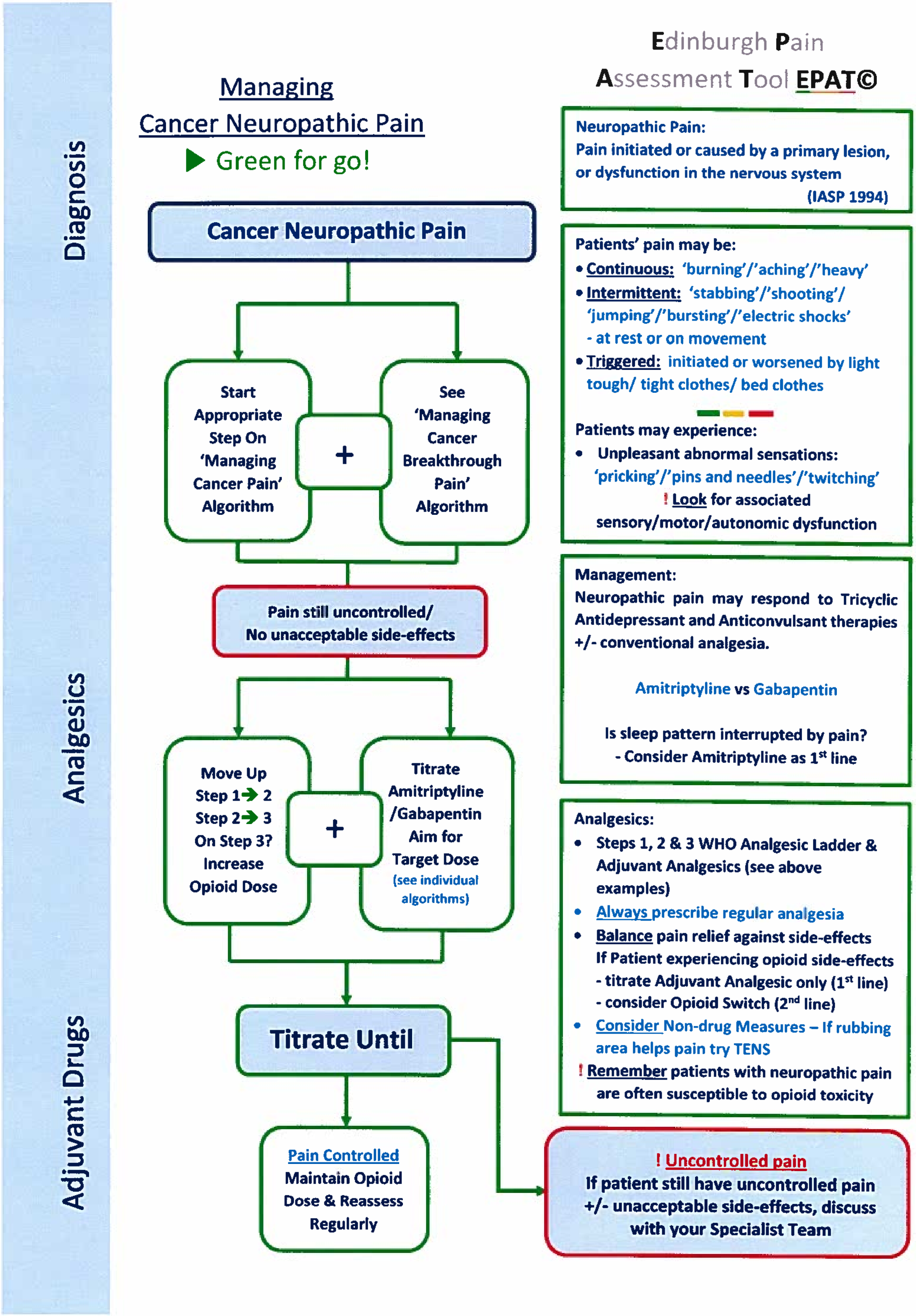
Diagnosis
Analgesics
Adjuvant Drugs
Managing
Cancer Neuropathic Pain
Green for go!
Cancer Neuropathic Pain
Start Appropriate Step On 'Managing Cancer Pain' Algorithm
+
See 'Managing Cancer Breakthrough Pain' Algorithm
Pain still uncontrolled/
No unacceptable side-effects
Move Up
Step 1→ 2
Step 2→ 3
On Step 3?
Increase
Opioid Dose
+
Titrate Amitriptyline /Gabapentin Aim for Target Dose
(see individual algorithms)
Titrate Until
Pain Controlled
Maintain Opioid Dose & Reassess Regularly
Edinburgh Pain
Assessment Tool EPAT©
Neuropathic Pain:
Pain initiated or caused by a primary lesion, or dysfunction in the nervous system
(IASP 1994)
Patients' pain may be:
• Continuous: 'burning'/'aching'/'heavy'
• Intermittent: 'stabbing'/'shooting'/ 'jumping'/'bursting'/'electric shocks'
- at rest or on movement
• Triggered: initiated or worsened by light tough/ tight clothes/ bed clothes
Patients may experience:
• Unpleasant abnormal sensations: 'pricking'/'pins and needles'/'twitching'
! Look for associated sensory/motor/autonomic dysfunction
Management:
Neuropathic pain may respond to Tricyclic Antidepressant and Anticonvulsant therapies +/- conventional analgesia.
Amitriptyline vs Gabapentin
Is sleep pattern interrupted by pain?
- Consider Amitriptyline as 1st line
Analgesics:
• Steps 1, 2 & 3 WHO Analgesic Ladder & Adjuvant Analgesics (see above examples)
• Always prescribe regular analgesia
• Balance pain relief against side-effects
If Patient experiencing opioid side-effects
- titrate Adjuvant Analgesic only (1st line)
- consider Opioid Switch (2nd line)
• Consider Non-drug Measures – If rubbing area helps pain try TENS
! Remember patients with neuropathic pain are often susceptible to opioid toxicity
! Uncontrolled pain
If patient still have uncontrolled pain +/- unacceptable side-effects, discuss with your Specialist Team

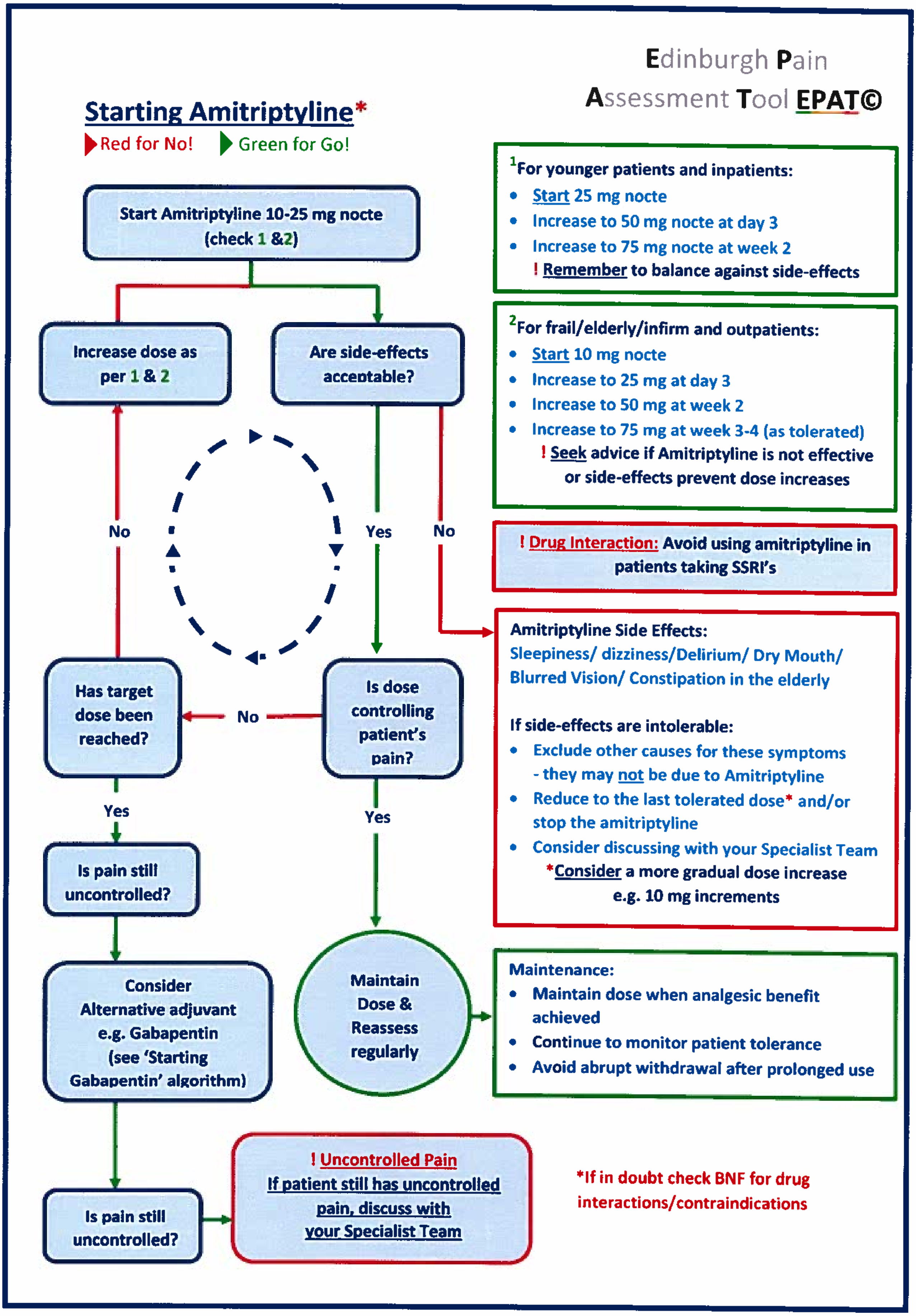
Edinburgh Pain Assessment Tool EPAT©
Starting Amitriptyline*
Red for No!
Green for Go!
Start Amitriptyline 10-25 mg nocte (check 1 &2)
Increase dose as per 1 & 2
Are side-effects acceptable?
No
Yes
No
Has target dose been reached?
No
Is dose controlling patient's pain?
Yes
Yes
Is pain still uncontrolled?
Consider Alternative adjuvant e.g. Gabapentin (see 'Starting Gabapentin' algorithm)
Maintain Dose & Reassess regularly
Is pain still uncontrolled?
! Uncontrolled Pain
If patient still has uncontrolled pain, discuss with your Specialist Team
1For younger patients and inpatients:
• Start 25 mg nocte
• Increase to 50 mg nocte at day 3
• Increase to 75 mg nocte at week 2
! Remember to balance against side-effects
2For frail/elderly/infirm and outpatients:
• Start 10 mg nocte
• Increase to 25 mg at day 3
• Increase to 50 mg at week 2
• Increase to 75 mg at week 3-4 (as tolerated)
! Seek advice if Amitriptyline is not effective or side-effects prevent dose increases
! Drug Interaction: Avoid using amitriptyline in patients taking SSRI's
Amitriptyline Side Effects:
Sleepiness/ dizziness/Delirium/ Dry Mouth/ Blurred Vision/ Constipation in the elderly
If side-effects are intolerable:
• Exclude other causes for these symptoms - they may not be due to Amitriptyline
• Reduce to the last tolerated dose* and/or stop the amitriptyline
• Consider discussing with your Specialist Team
*Consider a more gradual dose increase e.g. 10 mg increments
Maintenance:
• Maintain dose when analgesic benefit achieved
• Continue to monitor patient tolerance
• Avoid abrupt withdrawal after prolonged use
*If in doubt check BNF for drug interactions/contraindications

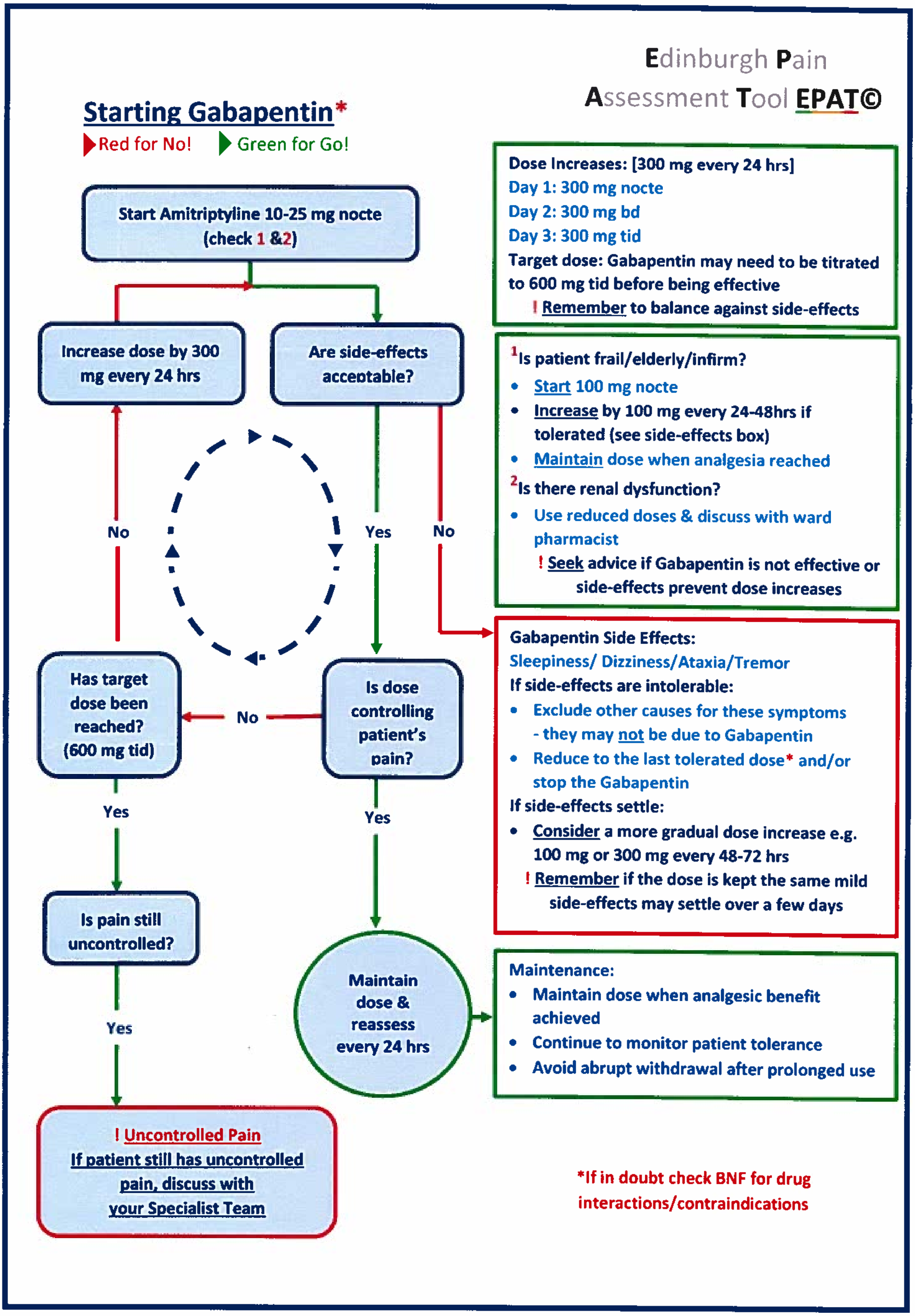
Edinburgh Pain Assessment Tool EPAT©
Starting Gabapentin*
Red for No!
Green for Go!
Start Amitriptyline 10-25 mg nocte (check 1 &2)
Increase dose by 300 mg every 24 hrs
Are side-effects acceptable?
No
Yes
No
Has target dose been reached? (600 mg tid)
No
Is dose controlling patient's pain?
Yes
Yes
Is pain still uncontrolled?
Yes
Maintain dose & reassess every 24 hrs
! Uncontrolled Pain
If patient still has uncontrolled pain, discuss with your Specialist Team
Dose Increases: [300 mg every 24 hrs]
Day 1: 300 mg nocte
Day 2: 300 mg bd
Day 3: 300 mg tid
Target dose: Gabapentin may need to be titrated to 600 mg tid before being effective
! Remember to balance against side-effects
1 Is patient frail/elderly/infirm?
Start 100 mg nocte
Increase by 100 mg every 24-48hrs if tolerated (see side-effects box)
Maintain dose when analgesia reached
2 Is there renal dysfunction?
Use reduced doses & discuss with ward pharmacist
! Seek advice if Gabapentin is not effective or side-effects prevent dose increases
Gabapentin Side Effects:
Sleepiness/ Dizziness/Ataxia/Tremor
If side-effects are intolerable:
Exclude other causes for these symptoms - they may not be due to Gabapentin
Reduce to the last tolerated dose* and/or stop the Gabapentin
If side-effects settle:
Consider a more gradual dose increase e.g. 100 mg or 300 mg every 48-72 hrs
! Remember if the dose is kept the same mild side-effects may settle over a few days
Maintenance:
Maintain dose when analgesic benefit achieved
Continue to monitor patient tolerance
Avoid abrupt withdrawal after prolonged use
*If in doubt check BNF for drug interactions/contraindications
