# Supplementary material for: Cancer pain assessment and management: does an institutional approach individualise and reduce cost of care?
Source: BMJ Support Palliat Care. 2023 May 26;13(e3):e1258–64. doi: 10.1136/spcare-2022-003547 (PMC10850828; doi:10.1136/spcare-2022-003547)
Supplement: Supplementary data [file spcare-2022-003547supp002.pdf]

Appendix 2 (Cost Assessment)

Costs - Length of Stay (LoS)		
Source	Description	Cost/day
PSSRU 2018	Daycase	745
National schedule of reference costs (2017/18)	Daycase	742
* Decided for the “Daycase” costs as opposed to inpatient costs as the inpatient cost reflect a spell of care rather than a per diem cost.		

Costs - Interventions		
Intervention	Cost/Intervention	Assumption/Description
Local Nerve Block	160	Nerve Block or Destruction of Nerve, for Pain Management
Coeliac plexus block	160	Nerve Block or Destruction of Nerve, for Pain Management
Cordotomy	160	Assumption based on the price for a Local nerve block
External epidural	133	Epidural Under Image Control for Pain Management
External intrathecal	360	Assumption based on the price
Implanter intrathecal	360	Insertion of Intrathecal Drug Delivery Device for Treatment of Neurological Conditions, 19 years and over
TENS	10	Assumption (Price of Tens Unit at local Pharmacy £59.99 [25/01/2019])
Acupuncture	957	Acupuncture for Pain Management

Heat	5	Assumption (Price at local Pharmacy [25/01/2019])
Cold	2.5	Assumption (Price of Tens Unit at local Pharmacy £24.99 [25/01/2019])
Radiotherapy	103	*Deliver a Fraction of Treatment on a Megavoltage Machine £103
All costs are NHS reference costs 2017/18 unless stated otherwise		

Name	Dose	Unit	Cost per pack (BNF)	Cost/Unit	Assumption
Aspirin	5	ml		0.04	
Aspirin	10	ml		0.04	
Aspirin	75	mg	28 tablets 1.12	0.04	
Aspirin	150	mg		0.08	
Aspirin	300	mg	100 tablets 10.78	0.1078	
Aspirin	390	mg		0.2156	
Aspirin	600	mg		0.2156	
Paracetamol	1	g	100 tablets 2.50	0.025	
Paracetamol	1.5	g		0.0403	
Paracetamol	2	g		0.05	
Paracetamol	2.5	g		0.0653	
Paracetamol	3	g		0.075	
Paracetamol	5	g		0.1	
Paracetamol	500	mg	100 tablets 1.53	0.0153	
Paracetamol	1000	mg	100 tablets 2.50	0.025	
Co-codamol 8/500	8/500			0.024666667	

Co-codamol 8/500	1	tabs	30 tablets 0.74	0.024666667	
Co-codamol 8/500	1.5	tabs		0.037	
Co-codamol 8/500	2	tabs		0.049333333	
Co-codamol 15/500	15/500		100 tablets 3.56	0.0356	
Co-codamol 15/500	2	tabs		0.0712	
Co-dydramol	10	ml		0.198928571	
Co-dydramol	1	g	20mg/500mg tablets 56 for 5.57	0.198928571	
Co-dydramol	2	tabs		0.198928571	
Co-codamol 30/500	30/500		100 tablets 4.14	0.0414	
Co-codamol 30/500	30	mg		0.0414	
Co-codamol 30/500	500	mg		0.0414	
Co-codamol 30/500	1	tabs		0.0414	
Co-codamol 30/500	2	tabs		0.0828	
Codeine Phosphate	1	g		2.438	
Codeine Phosphate	30	g		2.438	
Codeine Phosphate	60	mcg	60 mg/ml 10 ampoules for 24.38	2.438	
Codeine Phosphate	15	mg	28 tablets for 0.74	0.026428571	
Codeine Phosphate	20	mg		0.031071429	£ from 30mg
Codeine Phosphate	22.5	mg		0.031071429	£ from 30mg
Codeine Phosphate	30	mg	28 tablets for 0.87	0.031071429	

Codeine Phosphate	45	mg		0.0575	
Codeine Phosphate	60	mg	28 tabs for 1.48	0.052857143	
Codeine Phosphate	90	mg		0.083928571	
Codeine Phosphate	120	mg		0.105714286	
Co-proxamol	2	tabs	100 tablets 1.53	0.0153	
Dihydrocodeine	2	tabs		0.061428571	
Dihydrocodeine	30	mg	28 tablets 0.86	0.030714286	
Dihydrocodeine	60	mg		0.061428571	£ from 30mg
Tramadol	5	mg		0.076666667	£ from 50mg
Tramadol	40	mg		0.076666667	£ from 50mg
Tramadol	50	mg	60 for 4.60	0.076666667	
Tramadol	75	mg	60 for 5.15	0.085833333	
Tramadol	100	mg	60 for 18	0.3	NHS indicative price
Tramadol	150	mg	60 for 23.28 (NHS indicative price)	0.388	
Tramadol	200	mg	60 for 31.04 (NHS ind.)	0.517333333	
Tramadol	250	mg		0.594	
Tramadol	500	mg		1.334666667	
Alfentanil	140	mcg		0.634	£ from 500mcg
Alfentanil	200	mcg		0.634	£ from 500mcg
Alfentanil	250	mcg		0.634	£ from 500mcg
Alfentanil	280	mcg		0.634	£ from 500mcg
Alfentanil	400	mcg		0.634	£ from 500mcg
Alfentanil	420	mcg		0.634	£ from 500mcg

Alfentanil	500	mcg	10 ampoule 6.34 (500 mcg/ml)	0.634	
Alfentanil	750	mcg		0.951	
Alfentanil	0.1	mg		0.634	£ from 500mcg
Alfentanil	0.15	mg		0.634	£ from 500mcg
Alfentanil	0.2	mg		0.634	£ from 500mcg
Alfentanil	0.5	mg		0.634	£ from 500mcg
Alfentanil	0.65	mg		0.951	£ from 750mcg
Alfentanil	0.8	mg		1.268	£ from 500mcg
Alfentanil	0.125	mg		1.268	£ from 500mcg
Alfentanil	0.4	mg		1.268	£ from 500mcg
Alfentanil	1	mg		1.268	£ from 500mcg
Alfentanil	1.2	mg		2.319	£ from 5mg
Alfentanil	1.5	mg		2.319	£ from 5mg
Alfentanil	10	mg		2.319	£ from 5mg
Alfentanil	10.5	mg		2.319	£ from 5mg
Alfentanil	12	mg		2.319	£ from 5mg
Alfentanil	12.5	mg		2.319	£ from 5mg
Alfentanil	15	mg		2.319	£ from 5mg
Alfentanil	18	mg		2.319	£ from 5mg
Alfentanil	2	mg		2.319	£ from 5mg
Alfentanil	2.125	mg		2.319	£ from 5mg
Alfentanil	2.25	mg		2.319	£ from 5mg
Alfentanil	20	mg		2.319	£ from 5mg
Alfentanil	22.5	mg		2.319	£ from 5mg
Alfentanil	25	mg		2.319	£ from 5mg

Alfentanil	3	mg		2.319	£ from 5mg
Alfentanil	35	mg		2.319	£ from 5mg
Alfentanil	4	mg		2.319	£ from 5mg
Alfentanil	42	mg		2.319	£ from 5mg
Alfentanil	420	mg		2.319	£ from 5mg
Alfentanil	5	mg	5mg/ml 10 amploules 23.19	2.319	£ from 5mg
Alfentanil	6	mg		4.638	£ from 5mg
Alfentanil	8	mg		4.638	£ from 5mg
Targinact 5/2.5	1	tabs	28 tablets 21.16	0.755714286	
Targinact 5/2.5	5	tabs		3.778571429	£ from5/2.5
Targinact 10/5	1	tabs	56 tablets 42.32	0.755714286	
Targinact 10/5	5	tabs		3.778571429	£ from 10/5
Targinact 20/10	1	tabs	56 tablets 84.62	1.511071429	
Targinact 20/10	2	tabs		3.022142857	
Targinact 20/10	3	tabs		4.533214286	
Targinact 40/20	1	tabs	56 tablets 169.28	3.022857143	
Buprenorphine	5	mcg		0.1008	£ from 200mcg
Buprenorphine	10	mcg		0.1008	£ from 200mcg
Buprenorphine	12	mcg		0.1008	£ from 200mcg
Buprenorphine	20	mcg		0.1008	£ from 200mcg
Buprenorphine	25	mcg		0.1008	£ from 200mcg
Buprenorphine	30	mcg		0.1008	£ from 200mcg
Buprenorphine	37.5	mcg		0.1008	£ from 200mcg
Buprenorphine	50	mcg		0.1008	£ from 200mcg
Buprenorphine	62	mcg		0.1008	£ from 200mcg

Buprenorphine	70	mcg		0.1008	£ from 200mcg
Buprenorphine	75	mcg		0.1008	£ from 200mcg
Buprenorphine	100	mcg		0.1008	£ from 200mcg
Buprenorphine	200	mcg	50 tablets 5.04	0.1008	
Buprenorphine	400	mcg		0.2016	£ from 200mcg
Diamorphine	1.5	mg		2.8	£ from 5mg
Diamorphine	2.5	mg		2.8	£ from 5mg
Diamorphine	4	mg		2.8	£ from 5mg
Diamorphine	5	mg	5 ampoule 14.00	2.8	
Diamorphine	7.5	mg		2.094	£ from 10mg
Diamorphine	10	mg	5 ampoule 10.47	2.094	
Diamorphine	15	mg		2.292	£ from 30mg
Diamorphine	20	mg		2.292	£ from 30mg
Diamorphine	25	mg		2.292	£ from 30mg
Diamorphine	30	mg	5 ampoule 11.46	2.292	
Diamorphine	35	mg		4.584	£ from 30mg
Diamorphine	40	mg		4.584	£ from 30mg
Diamorphine	45	mg		4.584	£ from 30mg
Diamorphine	50	mg		4.584	£ from 30mg
Diamorphine	55	mg		4.584	£ from 30mg
Diamorphine	60	mg		4.584	£ from 30mg
Diamorphine	65	mg		10.022	£ from 100mg
Diamorphine	80	mg		10.022	£ from 100mg
Diamorphine	90	mg		10.022	£ from 100mg
Diamorphine	100	mg	5 ampoule 50.11	10.022	
Diamorphine	115	mg		20.044	£ from 100mg

Diamorphine	120	mg		20.044	£ from 100mg
Diamorphine	150	mg		20.044	£ from 100mg
Diamorphine	200	mg		20.044	£ from 100mg
Diamorphine	225	mg		40.342	£ from 500mg
Diamorphine	250	mg		40.342	£ from 500mg
Diamorphine	267	mg		40.342	£ from 500mg
Diamorphine	400	mg		40.342	£ from 500mg
Diamorphine	500	mg	5 ampoule 201.71	40.342	
Diamorphine	650	mg		60.386	Mix ampoules
Diamorphine	900	mg		80.684	Mix ampoules
Fentanyl	5	mg	TD 50mcg/h 5patch for 33.66	6.732	
Fentanyl	7.5	mg		3.598	
Fentanyl	25	mg	TD 25mcg/h 5patch for 17.99	3.598	
Fentanyl	37	mg	TD 75mcg/h 5patch for 46.99	9.398	
Fentanyl	37.5	mg	TD 75mcg/h 5patch for 46.99	9.398	
Fentanyl	100	mg	Sublingual tablet 10 tablets 49.99	4.999	£ from 400mg
Fentanyl	200	mg	Sublingual tablet 10 tablets 49.99	4.999	£ from 400mg
Fentanyl	400	mg	Sublingual tablet 10 tablets 49.99	4.999	

Fentanyl	6	mcg	Topical 1 Spray 50mcg 6 doses 35.70	5.95	
Fentanyl	12	mcg		11.9	
Fentanyl	12.5	mcg		11.9	
Fentanyl	20	mcg	50mcg/ml 10 ampoules 14.17	14.17	
Fentanyl	25	mcg		17.85	
Fentanyl	36	mcg		35.7	
Fentanyl	37	mcg		35.7	
Fentanyl	37.5	mcg		35.7	
Fentanyl	40	mcg	50mcg/ml 10ampoules 14.17	14.17	
Fentanyl	50	mcg	50mcg/ml 10ampoules 14.17	14.17	
Fentanyl	62	mcg		5.95	£ from 100mcg
Fentanyl	72	mcg		5.95	£ from 100mcg
Fentanyl	75	mcg		5.95	£ from 100mcg
Fentanyl	87	mcg		5.95	£ from 100mcg
Fentanyl	100	mcg	100mcg per dose -6 dose 35.70	5.95	
Fentanyl	125	mcg		5.95	
Fentanyl	150	mcg	Subcutaneous infusion	15	

			50mcg/ml 5.00(NHS ind.)		
Fentanyl	175	mcg	200mcg per dose 6 dose 35.70	5.95	
Fentanyl	200	mcg	Buccal 28 tablets 139.72	4.99	
Fentanyl	225	mcg	lozenge 400mcg 3 lozenge 21.05	7.016666667	
Fentanyl	250	mcg	subcutaneous infusion	25	
Fentanyl	275	mcg		11.9	
Fentanyl	300	mcg	subcutaneous infusion	30	
Fentanyl	350	mcg	lozenge 400mcg 3 lozenge 21.05	7.016666667	
Fentanyl	400	mcg	subcutaneous infusion	40	
Fentanyl	600	mcg	subcutaneous infusion	60	
Fentanyl	800	mcg	subcutaneous infusion	80	
Fentanyl	1200	mcg	600mcg Sublingual 30 tablets 149.70	9.98	
Hydromorphone	1.3	mg		0.374642857	£ from 2mg
Hydromorphone	2	mg	56 capsules 20.98	0.374642857	

Hydromorphone	2.5	mg		0.513392857	£ from 4mg
Hydromorphone	2.6	mg		0.513392857	£ from 4mg
Hydromorphone	4	mg	56 capsules 28.75	0.513392857	
Hydromorphone	5.2	mg		1.001428571	£ from 8mg
Hydromorphone	6	mg		1.001428571	£ from 8mg
Hydromorphone	6.5	mg		1.001428571	£ from 8mg
Hydromorphone	7.6	mg		1.001428571	£ from 8mg
Hydromorphone	7.8	mg		1.001428571	£ from 8mg
Hydromorphone	8	mg	56 capsules 56.08	1.001428571	
Hydromorphone	14	mg		1.902321429	£ from 14mg
Hydromorphone	16	mg	56 capsules 106.53	1.902321429	
Hydromorphone	18	mg		2.853928571	£ from 24mg
Hydromorphone	20	mg		2.853928571	£ from 24mg
Hydromorphone	24	mg	56 capsules 159.82	2.853928571	
Methadone	70	ml	50mg/ml 10 ampoules 17.72 (take 2)	3.544	
Methadone	10	mg	5mg tables 50 for 2.84	5.68	
Methadone	15	mg		8.52	Mix dose
Methadone	20	mg		11.36	Mix dose
Methadone	30	mg		17.04	Mix dose
Methadone	56	mg		31.24	Mix dose
Methadone	60	mg		34.08	Mix dose

Methadone	65	mg		36.92	Mix dose
Morphine	1	g		5.78	Mix dose
Morphine	1.2	g		6.936	Mix dose
Morphine	7.5	g		43.35	Mix dose
Morphine	2.5	ml	1 mg/ml 5.78	14.45	
Morphine	5	ml		28.9	Mix dose
Morphine	10	ml		57.8	Mix dose
Morphine	1	mg		0.054833333	£ from 5mg
Morphine	1.25	mg		0.054833333	£ from 5mg
Morphine	2	mg		0.054833333	£ from 5mg
Morphine	2.5	mg		0.054833333	£ from 5mg
Morphine	5	mg	60 for 3.29	0.054833333	
Morphine	6	mg		0.086666667	£ from 10mg
Morphine	7.5	mg		0.086666667	£ from 10mg
Morphine	8	mg		0.086666667	£ from 10mg
Morphine	10	mg	60 for 5.20	0.086666667	
Morphine	12.5	mg		0.207833333	£ from 30mg
Morphine	15	mg		0.207833333	£ from 30mg
Morphine	17.5	mg		0.207833333	£ from 30mg
Morphine	20	mg		0.207833333	£ from 30mg
Morphine	25	mg		0.207833333	£ from 30mg
Morphine	30	mg	60 for 12.47	0.207833333	
Morphine	35	mg		0.405333333	£ from 60mg
Morphine	39	mg		0.405333333	£ from 60mg
Morphine	40	mg		0.405333333	£ from 60mg
Morphine	45	mg		0.405333333	£ from 60mg

Morphine	50	mg		0.405333333	£ from 60mg
Morphine	55	mg		0.405333333	£ from 60mg
Morphine	60	mg	60 for 24.32	0.405333333	
Morphine	65	mg		0.641666667	£ from 100mg
Morphine	70	mg		0.641666667	£ from 100mg
Morphine	75	mg		0.641666667	£ from 100mg
Morphine	80	mg		0.641666667	£ from 100mg
Morphine	90	mg		0.641666667	£ from 100mg
Morphine	100	mg	60 for 38.50	0.641666667	
Morphine	110	mg		1.355666667	£ from 200mg
Morphine	120	mg		1.355666667	£ from 200mg
Morphine	125	mg		1.355666667	£ from 200mg
Morphine	130	mg		1.355666667	£ from 200mg
Morphine	140	mg		1.355666667	£ from 200mg
Morphine	150	mg		1.355666667	£ from 200mg
Morphine	160	mg		1.355666667	£ from 200mg
Morphine	170	mg		1.355666667	£ from 200mg
Morphine	180	mg		1.355666667	£ from 200mg
Morphine	200	mg	60 for 81.34	1.355666667	
Morphine	210	mg		1.442333333	Mix dose
Morphine	225	mg		1.5635	Mix dose
Morphine	230	mg		1.5635	Mix dose
Morphine	240	mg		1.761	Mix dose
Morphine	250	mg		1.761	Mix dose
Morphine	260	mg		1.761	Mix dose
Morphine	290	mg		1.997333333	£ from 300mg

Morphine	300	mg		1.997333333	
Oxycodone	5	ml	PO 5mg/ml solution 250ml for 9.71	0.1942	
Oxycodone	10	ml		0.3884	£ from 5ml
Oxycodone	15	ml		0.5826	£ from 5ml
Oxycodone	20	mcg		3.2	assume 20mg
Oxycodone	1	mg		0.223571429	£ from 5mg
Oxycodone	1.5	mg		0.223571429	£ from 5mg
Oxycodone	1.5	mg		0.223571429	£ from 5mg
Oxycodone	2	mg		0.223571429	£ from 5mg
Oxycodone	2.5	mg		0.223571429	£ from 5mg
Oxycodone	3	mg		0.223571429	£ from 5mg
Oxycodone	3.75	mg		0.223571429	£ from 5mg
Oxycodone	4	mg		0.223571429	£ from 5mg
Oxycodone	5	mg	PO 28 tablets 6.26	0.223571429	
Oxycodone	6.26	mg		1.6	£ from 10mg
Oxycodone	7	mg		1.6	£ from 10mg
Oxycodone	7.5	mg		1.6	£ from 10mg
Oxycodone	8	mg		1.6	£ from 10mg
Oxycodone	10	mg	SCSI 10mg/ml 5 ampoule 8.00	1.6	
Oxycodone	12.5	mg		4	£ from 15mg
Oxycodone	15	mg	SCSI 10mg/ml 5 ampoule 8.00	4	

Oxycodone	17	mg		3.2	£ from 20mg
Oxycodone	20	mg	SCSI 20mg/2ml 5 ampoule 16.00	3.2	
Oxycodone	22.5	mg		7.2	£ from 25mg
Oxycodone	30	mg		4.8	Mix dose
Oxycodone	35	mg		1.789107143	£ from 40mg
Oxycodone	40	mg	PO 56 tablets 100.19	1.789107143	
Oxycodone	45	mg		14.02	£ from 50mg
Oxycodone	50	mg	SCSI 50mg/1ml 5ampoule 70.10	14.02	
Oxycodone	55	mg		2.723035714	£ from 60mg
Oxycodone	60	mg	PO 56tablest 152.49	2.723035714	
Oxycodone	65	mg		3.578392857	£ from 80mg
Oxycodone	70	mg		3.578392857	£ from 80mg
Oxycodone	75	mg		3.578392857	£ from 80mg
Oxycodone	80	mg	PO 56 tablets 200.39	3.578392857	
Oxycodone	85	mg		22.82	Mix dose
Oxycodone	90	mg	SCSI	20.42	Mix dose
Oxycodone	100	mg	SCSI	28.04	Mix dose
Oxycodone	110	mg	PO 56 tablets 305.02	5.446785714	
Oxycodone	120	mg	SCSI	31.24	Mix dose
Oxycodone	125	mg		11.57821429	Mix dose

Oxycodone	130	mg		11.57821429	Mix dose
Oxycodone	135	mg		34.44	Mix dose
Oxycodone	140	mg	SCSI	34.44	Mix dose
Oxycodone	150	mg	SCSI	42.06	Mix dose
Oxycodone	160	mg		27.23392857	Mix dose
Oxycodone	180	mg		27.23392857	Mix dose
Oxycodone	200	mg		27.23392857	Mix dose
Oxycodone	210	mg		27.23392857	Mix dose
Oxycodone	240	mg		27.23392857	Mix dose
Oxycodone	260	mg		27.23392857	Mix dose
Oxycodone	280	mg		27.23392857	Mix dose
Oxycodone	320	mg		27.23392857	Mix dose
Oxycodone	360	mg		27.23392857	Mix dose
Oxycodone	400	mg		27.23392857	Mix dose
Oxycodone	460	mg		27.23392857	Mix dose
Oxycodone	560	mg	PO	27.23392857	Mix dose
Clonidine	25	mcg	112 tablets 3.90	0.034821429	
Clonidine	4.8	mg	100mcg tablets 100 for 8.04	0.402	
Bupivacaine	4	ml	5mg/ml 10 ampoule 16.15	1.615	
Levobupivacaine	7	ml		3.23	£ from 10ml
Levobupivacaine	10	ml	5mg/ml 10 ampoule 16.15	3.23	
Lidocaine	10	ml		0.993333333	

Lidocaine	200	mg	20mg/ml 10 ampoule 19.50 (all needed)	19.5	
Lidocaine Patch	100	mcg	50mg per 1g 30plaster 72.40	4.826666667	£ from 1 patch
Lidocaine Patch	1	patch		4.826666667	
Lidocaine Patch	1.5	patch		7.24	
Lidocaine Patch	2	patch		9.653333333	
Lidocaine Patch	3	patch		14.48	
Lidocaine Patch	6	patch		28.96	
Lidocaine Gel	1	ml	20 mg per1g 30 for 2.98	0.099333333	
Lidocaine Gel	6	ml		0.596	Mix dose
Lidocaine Gel	1	n/a		0.099333333	£ from 1ml
Lidocaine Spray	1	n/a	10mg per 1 actuation 50actuations 6.29	0.1258	
Benzydamine	1	n/a	1.5mg/ml 30ml 3.97	1.323333333	
Benzydamine	10	ml		1.323333333	Mix dose
Benzydamine	15	ml		1.985	Mix dose
Carbamazepine	100	mg	84 tablet 2.07 (Hopital)	0.024642857	
Carbamazepine	200	mg	84 tablet 3.83	0.045595238	
Carbamazepine	300	mg		0.070238095	Mix dose
Carbamazepine	400	mg	56 tablet 5.02	0.089642857	

Clonazepam	500	mcg	100 tablets 28.02	0.2802	
Clonazepam	1	mg		0.5604	£ from 500mcg
Clonazepam	1.5	mg		0.8406	£ from 500mcg
Clonazepam	2	mg	100 tablets 29.43	0.2943	
Phenytoin	72	mg		0.511071429	£ from 75mg
Phenytoin	75	mg	50mg 28 capsules 7.07/ 25mg capsules 7.24	0.511071429	
Phenytoin	100	mg	28 tablets 11.76	0.42	
Phenytoin	150	mg		0.6725	Mix dose
Phenytoin	200	mg		0.84	£ from 100mg
Phenytoin	300	mg	28 capsules 9.11	0.325357143	
Phenytoin	350	mg		0.577857143	Mix dose
Phenytoin	500	mg		1.165357143	Mix dose
Phenytoin	1000	mg		1.396071429	Mix dose
Sodium Valproate	200	mg	100 tablets 11.65	0.1165	
Sodium Valproate	300	mg	100 tablets 17.47	0.1747	
Sodium Valproate	400	mg		0.233	Mix dose
Sodium Valproate	600	mg		0.3495	Mix dose
Sodium Valproate	800	mg		0.466	Mix dose
Sodium Valproate	1200	mg		0.6988	Mix dose
Gabapentin	100	g		0.0429	Mix dose
Gabapentin	1.2	g		0.2789	Mix dose
Gabapentin	100	mg	100 capsules 1.91	0.0191	
Gabapentin	150	mg		0.0429	£ from 300mg
Gabapentin	200	mg		0.0429	£ from 300mg

Gabapentin	233.3	mg		0.0429	£ from 300mg
Gabapentin	300	mg	100 capsules 4.29	0.0429	
Gabapentin	400	mg	100 capsules 3.91	0.0391	
Gabapentin	500	mg		0.0638	£ from 600mg
Gabapentin	600	mg	100 tablets 6.38	0.0638	
Gabapentin	700	mg		0.2398	£ from 800mg
Gabapentin	800	mg	100 tablets 23.98	0.2398	
Gabapentin	900	mg		0.2589	Mix dose
Gabapentin	1200	mg		0.2789	Mix dose
Pregabalin	25	mg	56 capsules 3.43	0.06125	
Pregabalin	50	mg	56 capsules 4.08	0.072857143	
Pregabalin	75	mg	56 capsules 3.18	0.056785714	
Pregabalin	100	mg	84 capsules 4.64	0.055238095	
Pregabalin	125	mg		0.116488095	Mix dose
Pregabalin	150	mg	56 capsules 3.44	0.061428571	
Pregabalin	200	mg	84 capsules 5.52	0.065714286	
Pregabalin	225	mg	56 capsules 3.48	0.068571429	
Pregabalin	250	mg		0.138571429	Mix dose
Pregabalin	300	mg	56 capsules 4.26	0.076071429	
Pregabalin	325	mg		0.137321429	Mix dose
Pregabalin	400	mg		0.131428571	Mix dose
Pregabalin	600	mg		0.152142857	Mix dose
Pregabalin	900	mg		0.228214286	Mix dose
Amitriptyline	10	mg	28 tablets 0.91	0.0325	
Amitriptyline	15	mg		0.025714286	£ from 25mg
Amitriptyline	20	mg		0.025714286	£ from 25mg

Amitriptyline	25	mg	28 tablets 0.72	0.025714286	
Amitriptyline	30	mg		0.086785714	£ from 50mg
Amitriptyline	37.5	mg		0.086785714	£ from 50mg
Amitriptyline	40	mg		0.086785714	£ from 50mg
Amitriptyline	50	mg	28 tablets 2.43	0.086785714	
Amitriptyline	60	mg		0.119285714	Mix dose
Amitriptyline	75	mg		0.1125	Mix dose
Amitriptyline	100	mg		0.173571429	Mix dose
Amitriptyline	150	mg		0.260357143	Mix dose
Amitriptyline	200	mg		0.347142857	Mix dose
Nortriptyline	10	mg	100 tablets 3.53	0.0353	
Nortriptyline	20	mg		0.0706	Mix dose
Nortriptyline	25	mg	100 tablets 4.55	0.0455	
Nortriptyline	30	mg		0.1059	Mix dose
Nortriptyline	40	mg		0.1412	Mix dose
Duloxetine	30	mg	28 capsules 2.19	0.078214286	
Duloxetine	60	mg	28 capsules 4.12	0.147142857	
Citalopram	10	mg	28 tablets 0.83	0.029642857	
Citalopram	20	mg	28 tablets 1.00	0.035714286	
Citalopram	30	mg		0.065357143	Mix dose
Citalopram	40	mg	28 tablets 0.97	0.034642857	
Citalopram	60	mg		0.070357143	Mix dose
Fluoxetine	20	mg	30 capsule 0.61	0.020333333	
Fluoxetine	40	mg	30 capsule 1.80	0.06	
Fluoxetine	60	mg	30 capsule 6.01	0.200333333	
Paroxetine	20	mg	30 tablets 1.21	0.040333333	

Paroxetine	30	mg	30 tablets 2.65	0.088333333	
Paroxetine	40	mg	30 tablets 19.56	0.652	
Paroxetine	60	mg		0.692333333	Mix dose
Sertraline	50	mg	28 tablets 0.76	0.027142857	
Sertraline	100	mg	28 tablets 1.00	0.035714286	
Mirtazapine	15	mg	28 tablets 1.48	0.052857143	
Mirtazapine	30	mg	28 tablets 1.09	0.038928571	
Mirtazapine	45	mg	28 tablets 1.52	0.054285714	
Mirtazapine	60	mg		0.077857143	Mix dose
Trazodone	50	mg	84 capsules 5.10	0.060714286	
Trazodone	100	mg	56 capsules 5.81	0.10375	
Trazodone	150	mg	28 tablets 5.06	0.180714286	
Trazodone	300	mg		0.361428571	Mix dose
Venlafaxine	75	mg	56 tablets 4.12	0.073571429	
Venlafaxine	120	mg		0.147142857	Mix dose
Venlafaxine	225	mg	28 tablets 33.60	1.2	
Clomipramine	100	mg	50mg capsules 28 for 1.56	0.111428571	
Dosulepin	25	mg	28 capsules 0.92	0.032857143	
Dosulepin	50	mg		0.065714286	Mix dose
Dosulepin	75	mg	28 tablets 1.20	0.042857143	
Baclofen	50	mg		0.066666667	Mix dose
Baclofen	10	mg	84 tablets 1.12	0.013333333	
Baclofen	20	mg		0.026666667	Mix dose
Buscopan	400	mcg	20mg/ml 10 ampoule 2.92	0.584	

Buscopan	600	mcg		0.876	Mix dose
Buscopan	10	mg	PO 56 tablets 3.00	0.053571429	
Buscopan	20	mg	PO	0.107142857	
Buscopan	40	mg	20mg/ml solution 10 ampoule 2.92	0.584	
Buscopan	60	mg		0.876	Mix dose
Buscopan	80	mg		1.168	Mix dose
Buscopan	90	mg		1.46	Mix dose
Buscopan	100	mg		1.46	Mix dose
Buscopan	120	mg		1.752	Mix dose
Buscopan	240	mg		3.504	Mix dose
Diazepam	1	mg		0.018571429	Mix dose
Diazepam	2	mg	28 tablets 0.52	0.018571429	
Diazepam	2.5	mg		0.02	£ from 5mg
Diazepam	4	mg		0.02	£ from 5mg
Diazepam	5	mg	28 tablets 0.56	0.02	
Diazepam	10	mg	28 tablets 0.62	0.022142857	
Diazepam	12.5	mg		0.042142857	£ from 40mg
Diazepam	15	mg		0.042142857	£ from 40mg
Diazepam	20	mg		0.044285714	£ from 40mg
Diazepam	40	mg	10mg/2ml 10ampoules 5.50	2.2	
Lorazepam	500	mcg		0.111071429	£ from 1mg
Lorazepam	0	tabs		0.111071429	£ from 1mg
Lorazepam	0.5	mg		0.111071429	£ from 1mg

Lorazepam	1	mg	28 tablets 3.11	0.111071429	
Lorazepam	2	mg		0.222142857	Mix dose
Lorazepam	4	mg		0.444285714	Mix dose
Nitrazempam	5	mg	30 tablets 5.76	0.192	
Nitrazempam	10	mg		0.384	£ from 5mg
Temazepam	2	mg		0.05214	£ from 10mg
Temazepam	10	mg	500 tablets 26.07	0.05214	
Temazepam	15	mg		0.10428	Mix dose
Temazepam	20	mg	250 tablets 15.45	0.0618	
Temazepam	30	mg		0.11394	Mix dose
Temazepam	40	mg		0.1236	Mix dose
Zopiclone	3.25	mg		0.029285714	£ from 3.75mg
Zopiclone	3.75	mg	28 tablets 0.82	0.029285714	
Zopiclone	7.25	mg		0.029642857	£ from 7.5mg
Zopiclone	7.5	mg	28 tablets 0.83	0.029642857	
Zopiclone	15	mg		0.059285714	£ from 3.75mg
Zopiclone	22.5	mg		0.088928571	£ from 3.75mg
Zolpidem	3.75	mg		0.028214286	£ from 5mg
Zolpidem	5	mg	28 tablets 0.79	0.028214286	
Zolpidem	7.5	mg		0.032142857	£ from 10mg
Zolpidem	10	mg	28 tablets 0.90	0.032142857	
Zolpidem	12	mg		0.060357143	Mix dose
Midazolam	2.5	ml		0.962	£ from 3mg
Midazolam	1	mg		0.962	£ from 3mg
Midazolam	1.25	mg		0.962	£ from 3mg
Midazolam	1.5	mg		0.962	£ from 3mg

Midazolam	2	mg	2mg/2ml 10 ampoule 5.00	0.5	
Midazolam	2.5	mg	PO 2.5ml/0.5ml 4 unit dose 82.00	20.5	
Midazolam	3	mg		0.962	
Midazolam	4	mg	5mg/5ml 10 ampoule 9.62	0.962	
Midazolam	5	mg	BUCCAL 5mg/1ml 4 unit dose 85.50	21.375	
Midazolam	10	mg		4.78	£ from 25.5mg
Midazolam	13	mg		4.78	£ from 25.5mg
Midazolam	15	mg		4.78	£ from 25.5mg
Midazolam	20	mg		4.78	£ from 25.5mg
Midazolam	25.5	mg	50mg/50ml 1 time 9.56	4.78	
Midazolam	30	mg		9.56	£ from 40mg
Midazolam	40	mg	50mg/50ml 1 time 9.56	9.56	
Midazolam	60	mg		9.05	£ from 70mg
Midazolam	70	mg	100 mg/50ml solution 9.05	9.05	
Clodronate	520	mg	60 tablet 114.44	1.907333333	
Clodronate	800	mg	60 tablet 146.43	2.4405	
Disodium Pamidronate	60	mg	60mg/20ml 1 dose	110	

Disodium Pamidronate	70	mg		165	£ from 90mg
Disodium Pamidronate	90	mg	90mg/30ml 1 dose	165	
Ibandronic Acid	50	mg	28 tablets 6.04	0.215714286	
Zoledronic Acid	2	mg		150	£ from 4mg
Zoledronic Acid	4	mg	4mg/100ml 1 dose 150	150	
Ketoprofen	1	n/a	2.5% Gel 100 gram 2.44	0.244	assume 10g/use
Dexamethasone	8	mcg		2.184	£ from 1mg
Dexamethasone	500	mcg	28 tablets 11.87	0.423928571	
Dexamethasone	0.4	mg	IV	2.184	
Dexamethasone	0.5	mg	IV	2.184	
Dexamethasone	1	mg	3.3mg/1ml 10 ampoule 21.84	2.184	
Dexamethasone	2	mg	50 tablets 8.10	0.162	
Dexamethasone	2.5	mg	3.3mg/1ml 10 ampoule 21.84	2.184	
Dexamethasone	3	mg	3.3mg/1ml 10 ampoule 21.84	2.184	
Dexamethasone	4	mg	50 tablets 85.50	1.71	
Dexamethasone	6	mg	PO	1.872	
Dexamethasone	8	mg	50 tablets 120	2.4	
Dexamethasone	10	mg		20	£ from 40mg
Dexamethasone	12	mg		20	£ from 40mg

Dexamethasone	16	mg		20	£ from 40mg
Dexamethasone	20	mg		20	£ from 40mg
Dexamethasone	40	mg	10 tablets 200	20	
Dexamethasone	80	mg		40	£ from 40mg
Hydrocortisone	1	n/a		0.909333333	£ from 10mg
Hydrocortisone	2.5	mg		0.909333333	£ from 10mg
Hydrocortisone	5	mg		0.909333333	£ from 10mg
Hydrocortisone	10	mg	30 tablets 27.28	0.909333333	
Hydrocortisone	20	mg	30 tablets 82.63	2.754333333	
Hydrocortisone	40	mg		5.508666667	Mix dose
Hydrocortisone	50	mg		6.418	Mix dose
Hydrocortisone	100	mg	IV 100mg 10 vials 9.17	0.917	
Hydrocortisone	150	mg		19.254	Mix dose
Prednisolone	1	n/a		0.02	£ from 1mg
Prednisolone	1	mg	28 tablest 0.56	0.02	
Prednisolone	2	mg		0.04	£ from 2.5mg
Prednisolone	2.5	mg	28 tablets 1.42	0.050714286	
Prednisolone	3	mg		0.06	Mix dose
Prednisolone	5	mg	30 tablets 0.95	0.031666667	
Prednisolone	7.5	mg		0.082380952	Mix dose
Prednisolone	10	mg	30 tablets 1.90	0.063333333	
Prednisolone	14	mg		0.126785714	£ from 20mg
Prednisolone	15	mg		0.126785714	£ from 20mg
Prednisolone	17.5	mg		0.126785714	£ from 20mg
Prednisolone	20	mg	28 tablets 3.55	0.126785714	

Prednisolone	25	mg	56 tablets 75.95	1.35625	
Prednisolone	30	mg	28 tablets 8.15	0.291071429	
Prednisolone	35	mg		0.417857143	Mix dose
Prednisolone	40	mg		0.253571429	Mix dose
Prednisolone	50	mg		2.7125	Mix dose
Prednisolone	60	mg		0.380357143	Mix dose
Prednisolone	65	mg		0.613809524	Mix dose
Prednisolone	70	mg		0.64547619	Mix dose
Prednisolone	80	mg		0.708928571	Mix dose
Prednisolone	100	mg		0.936547619	Mix dose
Methylprednisolone	1.5	mg		8.96	£ from 120mg
Methylprednisolone	60	mg		8.96	£ from 120mg
Methylprednisolone	120	mg	120mg/3ml 1 dose 8.96	8.96	
Celecoxib	3	g	300 mg	0.082666667	Assume dose
Celecoxib	100	mg	60 capsules 1.72	0.028666667	
Celecoxib	200	mg	30 capsules 1.62	0.054	
Naproxen	1	g		0.162857143	Mix dose
Naproxen	250	mg	56 tablets 4.29	0.076607143	
Naproxen	375	mg	56 tablets 22.16	0.395714286	
Naproxen	500	mg	56 tablets 4.56	0.081428571	
Parecoxib	20	mg	40mg powder 5 vial 28.34	5.668	
Piroxicam	1	n/a	0.5% Gel 112 gram 3.81	0.346363636	assume 10g/use

Diclofenac	1	n/a	1.16% Emulgel 100g 4.63	0.463	assume 10g/use
Diclofenac	2	tabs		0.059642857	£ from 25mg
Diclofenac	5	mg		0.059642857	£ from 25mg
Diclofenac	15	mg		0.059642857	£ from 25mg
Diclofenac	25	mg	PO 28 tablets 1.67	0.059642857	
Diclofenac	50	mg	PO 84 tablets 7.49	0.089166667	
Diclofenac	75	mg	PO 56 tablets 11.31	0.201964286	
Diclofenac	100	mg	PR 10 suppositories 3.64	0.364	
Diclofenac	150	mg		0.403928571	Mix dose
Diclofenac	400	mg		0.713333333	Mix dose
Etoricoxib	60	mg	28 tablets 13.99	0.499642857	
Ibuprofen	400	mcg		0.036309524	£ from 200mg
Ibuprofen	1	n/a	5%Gel 100 gra 2.16	0.0216	assume 10g/use
Ibuprofen	1	tabs		0.036309524	£ from 200mg
Ibuprofen	60	mg		0.036309524	£ from 200mg
Ibuprofen	200	mg	84 tablets 3.05	0.036309524	
Ibuprofen	265	mg	16 tablets 2.30	0.14375	
Ibuprofen	300	mg	24 capsule 4.20	0.175	
Ibuprofen	400	mg	60 tablets 4.90	0.081666667	

Ibuprofen	600	mg	60 tablets 3.60	0.06	
Ketorolac	10	mg		1.072	
Ketorolac	30	mg	30mg/1ml 5 ampoule 5.36	1.072	
Lumiracoxib	500	mg	200mg tablet 112 tablets 22.11	0.592232143	
Ketamine	5	mg		5.06	£ from 200mg
Ketamine	10	mg		5.06	£ from 200mg
Ketamine	15	mg		5.06	£ from 200mg
Ketamine	20	mg		5.06	£ from 200mg
Ketamine	25	mg		5.06	£ from 200mg
Ketamine	30	mg		5.06	£ from 200mg
Ketamine	40	mg		5.06	£ from 200mg
Ketamine	50	mg		5.06	£ from 200mg
Ketamine	60	mg		5.06	£ from 200mg
Ketamine	75	mg		5.06	£ from 200mg
Ketamine	80	mg		5.06	£ from 200mg
Ketamine	100	mg		5.06	£ from 200mg
Ketamine	120	mg		5.06	£ from 200mg
Ketamine	150	mg		5.06	£ from 200mg
Ketamine	200	mg	200mg/10ml 1vial 5.06	5.06	
Ketamine	250	mg		8.77	£ from 550mg
Ketamine	300	mg		8.77	£ from 550mg
Ketamine	400	mg		8.77	£ from 550mg
Ketamine	450	mg		8.77	£ from 550mg

Ketamine	550	mg	500mg/10ml 1 vial 8.77	8.77	
Aceclofenac	100	mg	60 tablets 9.63	0.1605	
Acitretin	40	mg	60 capsules 23.80	1.586666667	
Allopurinol	300	mg	28 tablets 1.00	0.035714286	
Alverine	60	mg	100 capsules 3.15	0.0315	
Apprepitant	80	mg	2 capsules 31.61	15.805	
Aprepitant	80	mg	3 capsules 31.61	15.805	
Aranesp (dorbopoetin)	30	mcg	4 injections 176.17	44.0425	
Atarax	10	mg	84 tablets 1.59	0.018928571	
Atarax	25	mg	28 tablets 0.83	0.029642857	
Bicalutamide	50	mg	28 tablets 12.66	0.452142857	
Bicalutamide	150	mg	28 tablets 8.91	0.318214286	
Carbocisteine	15	ml	250mg/5ml oral solution 300 ml 8.55	0.4275	
Casodex	50	mg	28 tablets 12.66	0.452142857	
Celecoxib	200	mg	30 capsules 1.62	0.054	
Chlordiazepoxide	5	mg	100 capsules 11.50	0.115	
Chlordiazepoxide	10	mg	100 capsules 17.80	0.178	
Chlordiazepoxide	20	mg		0.356	Mix dose
Chlorphenamine	10	mg	10mg/ml 5 ampoule 22.50	4.5	

Chlorpromazine	100	mg	28 tablets 35.46	1.266428571	
Ciclosporin	25	mg	30 capsules 18.37	0.612333333	
Ciclosporin	75	mg		1.837	Mix dose
Cipralex	20	mg	28 tablets 1.35	0.048214286	
Clexane	40	mg	10 injection 30.27	3.027	
Clexane	80	mg	10 injection 55.13	5.513	
Clomipramine	100	mg	28 capsules 1.56	0.111428571	
Cortisone Acetate	50	mg		1	No BNF cost assume £1
Cyclizine	30	mg		0.0635	£ from 50mg
Cyclizine	50	mg	100 tablets 6.35	0.0635	
Cyclizine	100	mg		0.127	£ from 50mg
Cyclizine	150	mg		0.1905	£ from 50mg
Cyclophosphamide	500	mg	1 vial 9.66	9.66	
Diethylstilbestrol	1	mg	28 tablets 208.00	7.428571429	
DOCUSATE	200	mg	100 mg capsules - 100 for 6.98	0.1396	
Domperidone	10	ml	1mg/ml - 200ml for 13.43	0.6715	
Domperidone	5	mg		0.03	£ from 10mg
Domperidone	10	mg	100 tablets 3.00	0.03	
Domperidone	15	mg		0.06	£ from 10mg
Domperidone	20	mg		0.06	£ from 10mg
Domperidone	50	mg		0.15	£ from 10mg
Dutasteride	500	mcg	30 capsule 3.94	0.131333333	
Entonox	1	n/a	available in Hosp.	0	

Escitalopram	5	mg	28 tablets 0.97	0.034642857	
Escitalopram	10	mg	28 tablets 1.05	0.0375	
Escitalopram	20	mg	28 tablets 1.35	0.048214286	
Etodolac	600	mg	30 tablets 15.50	0.516666667	
Exemastane	25	mg	30 tablets 6.50	0.216666667	
exemestane	25	mg	30 tablets 6.50	0.216666667	
Fentazin	4	mg		1	No BNF cost assume £1
Filgratism	480	mcg	5 injection 399.50	79.9	
Flupentixol	0.5	mg	60 tablets 2.88	0.048	
Flutamide	250	mg	84 tablets 106.24	1.264761905	
Folic Acid	5	mg	28 tablets 0.63	0.0225	
Gelclair	1	n/a	0.1% 10ml for 7.49	0.749	assume 1ml/use
Glucosamine	1500	mg	30 tablets 18.20	0.606666667	
Glycoperonium	2.4	mg	3 gram for 327.00	327	
Glycoperonium	200	mcg	10 ampoule 10.45	10.451	
Goserelin	3.6	mg	1 injection 65.00	65	
Granisetron	1	mg	10 tablets 40.79	4.079	
Haloperidol	500	mcg	28 tablets 22.79	0.813928571	
Haloperidol	0.5	mg	28 tablets 22.79	0.813928571	
Haloperidol	1	mg		1.627857143	£ from 0.5mg
Haloperidol	1.25	mg		0.52	£ from 1.5mg
Haloperidol	1.5	mg	28 tablets 14.56	0.52	
Haloperidol	2	mg		0.6075	£ from 5mg
Haloperidol	2.5	mg		0.6075	£ from 5mg

Haloperidol	3	mg		0.6075	£ from 5mg
Haloperidol	5	mg	28 tablets 17.01	0.6075	
Hydroxychloroquine	200	mg	60 tablets 3.96	0.066	
Hydroxyzine	25	mg	28 tablets 0.83	0.029642857	
Hyoscine	1.2	mg	PO? 600mcg/ml 10 ampoules 53.93	10.786	
Hyoscine butylbromide	20	mg	56 tablets (10mg) 3.00	0.107142857	
Hyoscine Hydrobromide	0.4	mg	20mg/ml 10 ampoule 2.92	0.584	
Hyoscine Hydrobromide	1	mg		1.46	Mix dose
Hyoscine Hydrobromide	1.2	mg		10.786	Mix dose
Hyoscine Hydrobromide	1.4	mg		11.00028571	Mix dose
Ibondronic Acid	50	mg	28 tablets 6.04	0.215714286	
Iscador	0.01	mg		1	No BNF cost assume £1
Lamotrigine	50	mg	56 tablets 4.77	0.085178571	
Lamotrigine	100	mg	56 tablets 7.91	0.14125	
Lamotrigine	150	mg		0.226428571	Mix dose
Lamotrigine	200	mg	56 tablets 10.59	0.189107143	
Lansoprazole	30	mg	28 capsules 0.91	0.0325	
Lenalidomide	25	mg	21 capsules 3426	163.1428571	

Letrozole	2.5	mg	14 tablets 0.89	0.063571429	
Levomeprazine	6	mg		0.241190476	£ from 12.5mg
Levomeprazine	6.25	mg		0.241190476	£ from 12.5mg
Levomeprazine	12.5	mg	25mg tablets - 84 tablets 20.26	0.241190476	
Levomepromazine	0.25	mg		0.241190476	£ from 12.5mg
Levomepromazine	1.25	mg		0.241190476	£ from 12.5mg
Levomepromazine	2.5	mg		0.241190476	£ from 12.5mg
Levomepromazine	3	mg		0.241190476	£ from 12.5mg
Levomepromazine	3.125	mg		0.241190476	£ from 12.5mg
Levomepromazine	6	mg		0.241190476	£ from 12.5mg
Levomepromazine	6.2	mg		0.241190476	£ from 12.5mg
Levomepromazine	6.25	mg		0.241190476	£ from 12.5mg
Levomepromazine	10	mg		0.241190476	£ from 12.5mg
Levomepromazine	12	mg		0.241190476	£ from 12.5mg
Levomepromazine	12.5	mg		0.241190476	£ from 12.5mg
Levomepromazine	18.75	mg		0.241190476	£ from 25mg
Levomepromazine	25	mg	25mg tablets - 84 tablets 20.26	0.241190476	
Levomepromazine	62.5	mg		0.723571429	
Lofepramine	70	mg	56 tablets 9.01	0.160892857	
Lofepramine	140	mg		0.321785714	Mix dose
Lofepramine	210	mg		0.482678571	Mix dose
Loperamide	2	mg	30 tablets 2.93	0.097666667	
Lorazopam	0.5	mg	1mg 28 tablets 3.11	0.111071429	

Maxalon	10	mg		1	No BNF cost assume £1
Mebeverine	135	mg	100 tablets 4.21	0.0421	
Medroxyprogesterone	400	mg	30 tablets 58.67	1.955666667	
Megace	60	mg		0.650666667	£ from 160mg
Megace	160	mg	30 tablets 19.52	0.650666667	
Megestrol	160	mg	30 tablets 19.52	0.650666667	
Meloxicam	7.5	mg	30 tablets 0.96	0.032	
Meloxicam	15	mg	30 tablets 0.99	0.033	
Methatroxate	2.5	mg	100 tablets 8.18	0.0818	
Methotrexate	15	mg	100 tablets 47.08	0.4708	
Metoclopramide	10	ml		0.018928571	£ from 10mg
Metoclopramide	5	mg		0.018928571	£ from 10mg
Metoclopramide	10	mg	28 tablets 0.53	0.018928571	
Metoclopramide	15	mg		0.037857143	£ from 10mg
Metoclopramide	20	mg		0.037857143	£ from 10mg
Metoclopramide	30	mg		0.056785714	£ from 10mg
Metoclopramide	40	mg		0.075714286	£ from 10mg
Metoclopramide	60	mg		2.223333333	£ from 80mg
Metoclopramide	70	mg		2.223333333	£ from 80mg
Metoclopramide	80	mg	10mg/20ml solution 12 ampoule 26.68	2.223333333	
Metoclopramide	120	mg		4.446666667	£ from 80mg
Metoclopramide	150	mg		4.446666667	£ from 80mg
Metoclopromide	10	mg	28 tablets 0.53	0.018928571	

Metoclopromide	15	mg		0.037857143	Mix dose
metrapozine	1	mg		1	No BNF cost assume £1
Metroclopramide	11	mg	10mg- 28 tablet 0.52	0.018928571	
Metrodopamide	10	mg	10mg- 28 tablet 0.53	0.018928571	
Nabumetone	500	mg	56 tablets 6.90	0.123214286	
Nefopam	30	mg	90 tablets 8.84	0.098222222	
Nefopam	60	mg		0.196444444	Mix dose
Norethisterone	10	mg	5mg - 90 tablets 4.21	0.093555556	
Nozinan	6.25	mg	25mg - 84 tablets 20.26	0.241190476	
Octreotide	300	mg	100mcg/ml 5 injections 30.34	18.204	
Olanzapine	20	mg	28 tablets 5.69	0.203214286	
OMEPRAZOLE	40	mg	7 capsules 0.54	0.077142857	
Ondansetron	10	ml		17.982	Mix dose
Ondansetron	4	mg	10 tablets 43.46	4.346	
Ondansetron	8	mg	10 tablets 85.43	8.543	
Ondansetron	16	mg	4mg/2ml solution 5 ampoule 29.97	23.976	
Ondansetron	40	mg	8mg/2ml solution 5 ampoule 59.95	59.95	
ondanstetron	8	mg	10 tablets 85.43	8.543	

Orabase	30	g	10 capsules 19.99	1.999	
Oxetacaine	5	mg		1	No BNF cost assume £1
Oxetacaine & Antacid	10	ml		1	No BNF cost assume £1
Oxycarbamazepine	75	mg		1	No BNF cost assume £1
Paclitaxel	234	mg	100mg/16.7ml 1 vial 200.35	601.05	
pamidronate	90	mg	90mg/10ml 1 vial 170.45	170.45	
Pethidine	6.25	mg		5.291	£ from 50mg
Pethidine	12.5	mg		5.291	£ from 50mg
Pethidine	16.25	mg		5.291	£ from 50mg
Pethidine	18.75	mg		5.291	£ from 50mg
Pethidine	25	mg		5.291	£ from 50mg
Pethidine	50	mg	50mg/5ml solution 10 ampoule 52.91	5.291	
Pethidine	150	mg	50 tablets 49.92	2.9952	
Pethidine	200	mg	50 tablets 49.92	3.9936	
Phosphate Sandoz	2	tabs	100 tablets 19.39	0.1939	
Piridoxine	80	mg	50mg tablets 28 for 15.52	1.108571429	
Piriton	10	mg	10mg/ml 5 ampoule 22.50	4.5	

Pizotifen	1.5	mg	28 tablets 1.27	0.045357143	
Priadel	200	mg	100 tablets 2.76	0.0276	
Priadel	600	mg		0.0828	£ from 200mg
Prochlorperazine	3	mg		0.030714286	£ from 5mg
Prochlorperazine	5	mg	84 tablets 2.58	0.030714286	
Prochlorperazine	10	mg		0.061428571	from 5mg
Progynova TS	100	mcg	12 patch 20.70	1.725	
Provera	400	mcg	30 tablets 58.67	1.955666667	
Quetiapine	25	mg	60 tablets 2.47	0.041166667	
Quinine	200	mg	28 tablets 1.49	0.053214286	
Quinine	300	mg	28 tablets 2.00	0.071428571	
Quinine Sulphate	200	mg	28 tablets 1.49	0.053214286	
Quinine Sulphate	300	mg	28 tablets 2.00	0.071428571	
Rameprazole	20	mg	28 tablets 1.36	0.048571429	
Rasberry Mucilage	10	ml		1	No BNF cost assume £1
Remedeine 30/500mg	2	tabs	56 tablets 6.82	0.243571429	
Risendronate	35	mg	4 tablets 0.76	0.19	
Sertraline syrup	100	mg	Syrup/ 28 tablets 1.00	0.035714286	
Sinutab	500	mg		1	No BNF cost assume £1
Sirolimus Liquid	3	mg	1mg/ml 60ml 162.41	8.1205	
Sodium Clodronate	520	mg	60 tablets 114.44	1.907333333	
Sodium Clodronate	800	mg	60 tablets 146.43	2.4405	

SODIUM PICOSULPHATE	10	mg	5mg/5ml 300ml 7.10	0.236666667	
Solifenacin	5	mg	30 tablets 35.91	1.197	
Spirolactone	100	mg	28 tablets 1.74	0.062142857	
Sucralfate	5	ml		1	No BNF cost assume £1
Sulfasalazine	1	g		0.1388	Mix dose
Sulfasalazine	500	mg	112 tablets 6.94	0.0694	
Sumatriptan	50	mg	6 tablets 4.05	0.675	
Tamoxifen	20	mg	30 tablets 2.35	0.078333333	
Tamsulosin	400	mg	30 capsules 3.87	0.129	
Testogel	50	mg	30 sachets 31.11	1.037	
Thalidomide	50	mg	28 capsules 298.48	10.66	
Thalidomide	200	mg		42.64	£ from 50mg
Thiamine	50	mg	100 tablets 4.35	0.0435	
Thyroxine	100	mg	Mcg/ 28 tablets 0.99	0.035357143	
Tolterodine	4	mg	2mg 56 tablets 2.78	0.049642857	
Tranexamic Acid	1	g		0.266666667	£ from 500mg
Tranexamic Acid	500	mg	500mg 60 tablets 8.00	0.133333333	
Trazadone	100	mg		1	No BNF cost assume £1

Trifluoperazine	10	mg	5mg tablets 112 tablets 134.89	2.40875	
Venlafaxine	75	mg	56 tablets 4.12	0.073571429	
Zaleplon	10	mg	7.5mg 28 tablets 0.83	0.059285714	
Zofran	4	mg	10 tablets 35.97	3.597	
Zoladex	10.8	mg	1 injection 235.00	235	
Zoledronic Acid	4	mg	4mg/100ml 1 bottle 174.14	174.14	
Zolpidem	10	mg	28 tablets 0.90	0.032142857	
